# Asymmetric plantar temperature downshifts are associated with atrial fibrillation and thromboembolic events: an observational post hoc analysis of the SmartPreventDiabeticFeet study

**DOI:** 10.1038/s43856-026-01811-3

**Published:** 2026-07-31

**Authors:** Antao Ming, Fidan Asadzade, Yunxin Zhang, Jaqueline Hötzsch, Gorre Sudha, Peter R. Mertens

**Affiliations:** 1 Key Laboratory of Laboratory Medicine, Ministry of Education, Institute of Genomic Medicine, School of Laboratory Medicine and Life Science, Wenzhou Medical University, Wenzhou, Zhejiang China; 2 University Clinic for Nephrology and Hypertension, Diabetes and Endocrinology, Otto-von-Guericke University Magdeburg, Magdeburg, Germany; 3 Department of Vascular Surgery, Beijing Jishuitan Hospital, Capital Medical University, Beijing, China

**Keywords:** Diabetes, Atrial fibrillation, Prognostic markers, Peripheral vascular disease, Thromboembolism

## Abstract

**Background:**

Cardiovascular and thromboembolic complications remain a major burden in diabetes. We aimed to determine whether peripheral thermometry is suited to detect plantar temperature downshifts (PTDs). Plantar infrared images (PIRIs) and sensor recordings were correlated with atrial fibrillation (AF) and thromboembolic events in patients with diabetes.

**Methods:**

A secondary analysis of the SmartPreventDiabeticFeet study including 239 patients monitored by PIRIs and sensor-equipped insoles (median follow-up 67.1 months) was conducted. In a structured telephone survey, histories of AF and thromboembolic events (stroke, pulmonary embolism [PE], peripheral arterial disease [PAD]) were captured from enrollment to post-intervention period. Patients with >30 days of temperature recordings (*n* = 118) were classified according to pre-specified PTD definitions, unsupervised clustering of bilateral plantar temperature variability, and plantar infrared image symmetry. Associations were tested using χ² or Fisher’s exact tests and multivariable logistic/Cox models.

****Results**:**

Our findings show that PTDs in sensor recordings as well as PIRI asymmetry are associated with a higher incidence of a composite of AF and thromboembolic events. Overall, 45 patients experience AF (*n* = 26), stroke (*n* = 6), PE (*n* = 4) or PAD (*n* = 11). Event rates are higher in patients with PTDs (25.8% vs 11.5%; *p* = 0.09; exploratory trend). For the elevated temperature variability cluster, the adjusted OR reaches 13.5 [95% CI 3.8–58.6] (34.0% vs 9.9%; *p* = 0.003; adjusted HR 13.3 [95% CI 1.5–120.4]). In individuals with plantar infrared image asymmetry, the likelihood is 48.6% compared to 6.2% in the symmetry group (*p* < 0.0001).

****Conclusions**:**

In patients with diabetes, plantar temperature downshifts are associated with thromboembolic risk constellation.

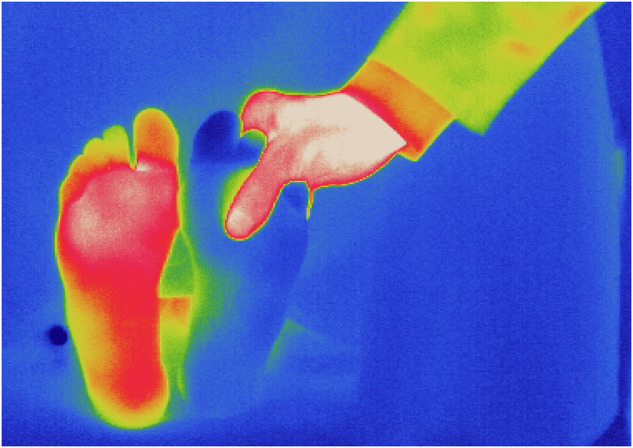

## Plain language summary

Individuals diagnosed with diabetes more often suffer from diseases such as heart attacks and heart arrhythmia. This can lead to further complications with stroke, pulmonary embolism, and insufficient blood supply to lower limbs. However, simple ways to identify risk constellations are limited. This study explores whether differences in plantar foot temperature occur and predict the onset of such complications. Foot temperature was quantified using infrared images or temperature sensor-equipped insoles in 239 patients during a long-term study lasting up to 75.9 months. A correlation of temperature imbalance with heart diseases such as irregular heartbeats and blood clotting diseases was found. Thus, foot temperature monitoring is a simple and noninvasive means to early-on identify individuals at risk for diabetes-associated diseases.

## Abstract (web summary)

Ming, Asadzade et al. identify patients with diabetes at risk for cardiovascular and thromboembolic events by peripheral thermography. Plantar infrared imaging and sensor-equipped insoles may pave the way toward early classification of patients for preventive anticoagulation.

## Introduction

The cardiovascular disease burden is notably high in patients with diabetes, with cardiovascular mortality rates accounting for approximately 50% of deaths^1^. A meta-analysis of 102 prospective studies found that diabetes doubles the risk of coronary heart disease (hazard ratio (HR) 2.00, 95% CI 1.83–2.19) and increases the risk of ischemic stroke by 127% (HR 2.27, 95% CI 1.95–2.65)^2^. The pathophysiology of this condition is linked with accelerated vascular aging due to poor glycemic control and endothelial cell activation^3^, immune cell recruitment to the vasculature and plaque formation^4,5^. The presence of diffuse narrowing of the arterial diameters within the extremities is attributed to a number of factors, including media hypertrophy, protruding plaques, or a combination of both^6^. Arterial hypertension is a common comorbidity of patients diagnosed with diabetes, frequently precipitated by the concomitant development of chronic kidney disease^7,8^. Arterial hypertension has been shown to result in a distinct pattern of damage to vascular wall structures through repetitive microtraumata and aneurysma formation^9,10^. Next to the vascular damage patterns with coronary insufficiency, cerebral and limb ischemia, there is an increased risk of thromboembolic events^11^. Arterial embolisms are most commonly caused by an enlarged left atrium, a condition that is frequently observed in patients suffering from cardiac dilatation due to mitral valve insufficiency or cardiac congestion with ischemic heart disease^12,13^. Patients suffering from an enlarged left atrium in conjunction with intermittent or permanent atrial fibrillation (AF) are predisposed to arterial thromboembolic events, including stroke and acute limb ischemia^14,15^. The annual incidence rate increases with age, reaching 39.3 per 1000 person-years in those older than 85 years^16,17^.

Previously, our group focused on plantar temperature changes that were recorded by insoles equipped with temperature sensors at six preselection sites^18–20^. The primary objective of the SmartPreventDiabeticFeet (SPDF) study was to evaluate the hypothesis that asymmetric temperature elevations may serve as an early indicator of diabetic foot ulcer development^19,20^. In the original SPDF trial, a mobile phone application-based alert system was established for diabetic foot prevention, incorporating a photographic documentation component, comprehensive instructions on offloading of the affected foot, and early consultation with attending healthcare professionals. All patients were diagnosed with diabetes. The exclusion criteria included an ankle-brachial index (ABI) below 0.5, and the majority of patients exhibited an ABI greater than 0.75.

During the study period, asymmetric temperature downshifts became apparent in a significant proportion of patients^19,20^. The aforementioned temperature downshifts were mostly transient, with a duration of several days to weeks, and often attained ambient temperature values that were concurrently recorded. Despite the extensive research conducted on temperature asymmetries, particularly those pertaining to elevated temperatures within the domain of diabetic foot ulcer prevention^18–22^, the clinical significance of temperature downshifts remains to be formally delineated. Prior work has shown that plantar temperature can exhibit rapid downshifts under physiological conditions such as standing/pressure exposure, consistent with perfusion-related changes at the skin surface and supporting the view that downshifts may indicate episodic distal hypoperfusion rather than a disease^18^. Because plantar skin temperature is tightly coupled to regional (sub-)cutaneous perfusion, episodic or asymmetric cooling may reflect transient reductions in distal blood flow^23^. In diabetes, such perfusion instability can plausibly arise from microvascular dysfunction and autonomic neuropathy with impaired vasomotor control and may be influenced by macrovascular disease^24,25^. Thromboembolic events cause immediate local hypoperfusion. Systematic analyses of such events have not been described in clinical studies before. It has to be emphasized that plantar temperature changes detected by thermography cannot distinguish underlying causes. This is reserved for additional examinations such as duplex ultrasound and angiography.

Here, we perform a post-hoc observational analysis to examine whether PTDs and plantar infrared imaging (PIRI) asymmetry are associated with AF and thromboembolic events (including pulmonary embolism (PE), stroke, or peripheral arterial disease (PAD)) in adults with diabetes. We show that plantar thermometry-derived asymmetry indicators are associated with a higher incidence of AF and thromboembolic events across complementary analytic approaches (OR 13.5 [95% CI 3.8–58.6]). Our findings from repeated temperature recordings suggest that short-lived vasomotor changes alone are unlikely to fully explain the observed PTDs. Instead, PTDs were consistent with episodic distal hypoperfusion. Thrombotic or emboligenic processes are one plausible contributor, but this remains additional confirmation using duplex ultrasound and angiography. The duration of PTDs and their concordance with infrared thermography support the interpretation of transient perfusion changes, while the underlying mechanism requires confirmation in prospective studies. These findings suggest that PTDs and PIRI asymmetry may identify a thromboembolic risk phenotype in high-risk patients with diabetes.

## Methods

### Participants

This study is an observational post-hoc exploratory analysis that utilizes data from the SPDF study cohort and combines them with reports obtained by a telephone questionnaire. The SPDF study was set up as a prospective, open-label, randomized, parallel-group clinical trial (German Clinical Trials Register: DRKS00013798) designed to investigate patients with diabetes mellitus and moderate-to-severe peripheral neuropathy at risk of diabetic foot ulceration^19,20^. The retrospective approach was chosen to combine long-term outcomes that are related to plantar temperature alterations during the study participation. Participants were recruited from the University Clinic for Nephrology and Hypertension, Diabetes and Endocrinology at the Otto-von-Guericke University Hospital Magdeburg.

### Ethics approval and consent to participate

The SPDF study was conducted in accordance with the Declaration of Helsinki and applicable ethical and regulatory requirements. The SPDF study protocol, informed consent forms, and questionnaires were reviewed and approved by the Ethics Committee of Otto-von-Guericke University Magdeburg on 8 January 2018 (file no. 176/17)^19^. Written informed consent was obtained from all participants prior to enrollment, including consent to be contacted during the follow-up period. The original SPDF clinical trial was registered in the German Clinical Trials Register (DRKS00013798; date of registration: 18 January 2018).

### Original SPDF intervention

The original SPDF study was designed to evaluate diabetic foot prevention rather than cardiovascular outcomes^19,20^. In brief, all participants received standardized foot-care education and scheduled follow-up, whereas participants in the intervention group additionally used sensor-equipped insoles and a cellphone application for home-based plantar temperature monitoring. The app-based alert system notified the study physician when predefined plantar temperature asymmetries occurred, prompting foot inspection, photo documentation, physician review, and, when clinically indicated, temporary foot offloading. The control group received standardized foot-care education and scheduled follow-up but did not receive app-based plantar temperature monitoring, automated alerts, or remote temperature-guided physician review.

### Baseline data collection

Baseline demographic and clinical data were collected, including age, sex, height, weight, body mass index (BMI), and maximum walking distance without assistance. The medical history was recorded, focusing on diabetes mellitus, thromboembolic diseases, peripheral neuropathy, prior foot lesions, deformities, and surgeries. Neuropathy severity was quantified by standardized clinical tests. Doppler ultrasound and ABI calculations were performed.

### Atrial fibrillation and thromboembolic outcomes

**Fig. 1:**
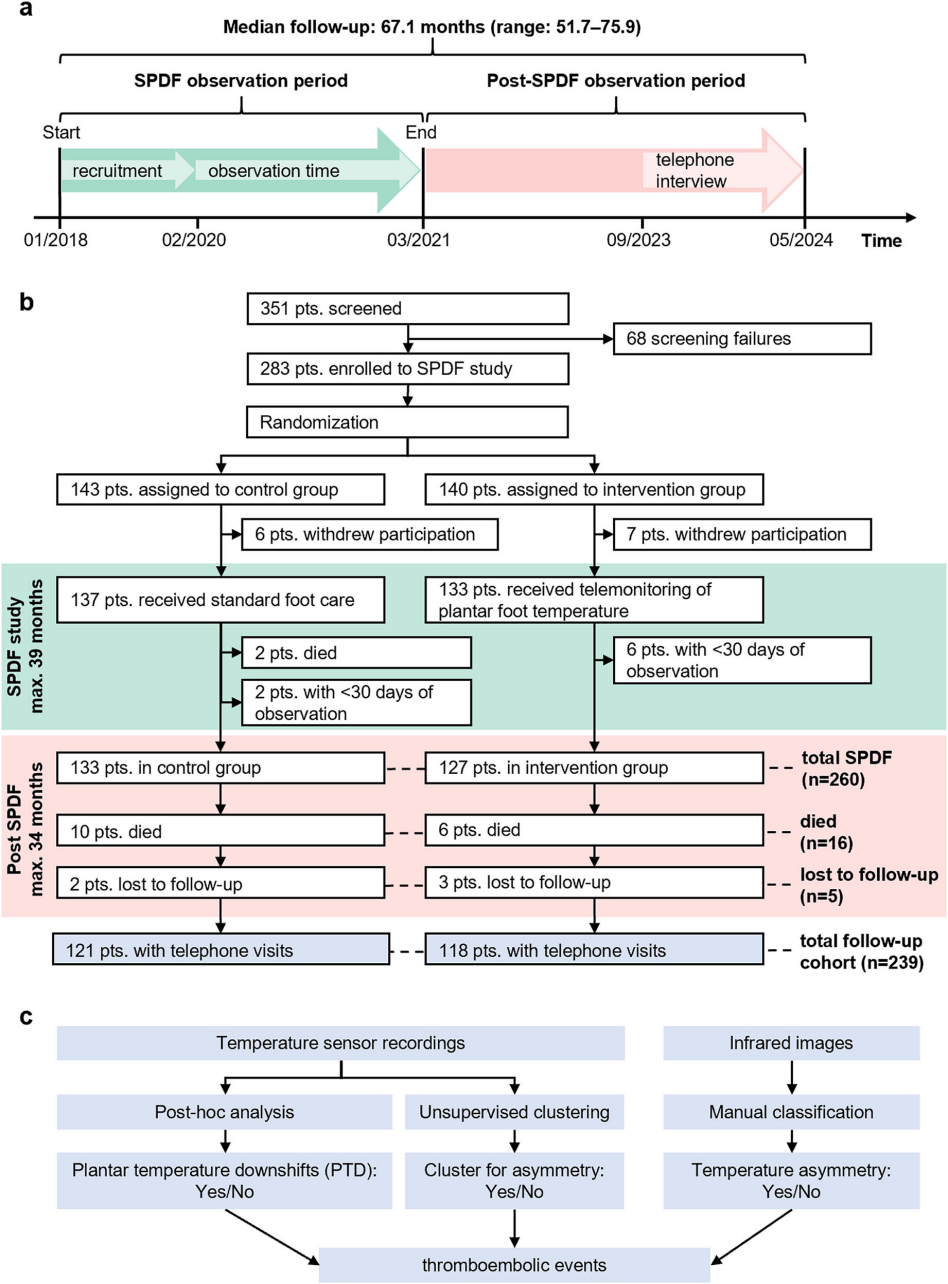
Study cohort and flow diagram (*n* = 239 patients). **a** Timeline illustrating the total follow-up duration. Atrial fibrillation and thromboembolic events were recorded continuously throughout the SPDF observation period (January 2018 to March 2021) and the post-SPDF observation period (March 2021 to May 2024). **b** Flowchart detailing the enrollment of participants, randomization, assignment to control and intervention groups, data availability, and data inclusion in the follow-up analyses. **c** Data analytic workflow: Sensor-derived plantar temperature time series were analyzed by manual identification of plantar temperature downshifts (PTDs; yes/no) and by unsupervised clustering of bilateral variability (asymmetry: yes/no), while plantar infrared images were manually classified as normal versus asymmetric. Risk strata from each approach were tested for association with atrial fibrillation and thromboembolic events.

From September 2023 to May 2024, participants were contacted via telephone in order to ascertain whether they had experienced any AF and thromboembolic events, including arrhythmias (e.g., AF), stroke, PE, and PAD, since the commencement of the study until the date of the interview. The precise temporal occurrence of the aforementioned events, in conjunction with the attendant clinical consequences - such as hospitalization, interventions, and modifications to medical management - was meticulously documented for the study and follow-up periods (January 2018 to May 2024; Fig. 1a).

### Infrared imaging of feet

Prior to the commencement of the study, plantar infrared images (PIRIs) were captured from the subjects’ feet with the objective of visualizing regions of hypo- or hyperthermia (Optris Pix Connect, Berlin). At the time of enrollment, no participant had a temperature difference between the right and left plantar sensors exceeding 0.5 °C. In the course of the regular follow-up visits within the study center, or on the occasion of additional visits instigated due to temperature asymmetries, infrared imaging was repeated. These images were utilized as resources to substantiate the observations of plantar temperature downshifts and were also analyzed separately.

### Plantar temperature recording and clustering analysis

During the SPDF study, participants in the intervention group were instructed to perform twice-daily plantar temperature measurements at intervals of at least four hours, with each session lasting three to five minutes by means of sensor-equipped insoles (Thorsis Technologies GmbH) and a mobile application developed by the University Clinic for Nephrology and Hypertension, Diabetes and Endocrinology^19^. These intervals were selected to capture diurnal variations in plantar temperature while minimizing participants' time burden. The mobile application transmitted plantar temperature data to the IQ-Trial Study Server located at the University Hospital, enabling monitoring by a physician with data access. Furthermore, participants were instructed to document their foot status and upload photographs thereof via the application.

For this observational post-hoc exploratory analysis, pre-processing of all collected plantar temperature data was performed to ensure data integrity and analytical reliability. This process involved the exclusion of invalid recordings, measurements that fell outside physiological body temperature ranges (<10 °C or >40 °C), duplicate entries with identical time stamps, and incomplete datasets (Supplementary Fig. 6). Sensors positioned at D1 were excluded from the analyses due to recurring measurement inconsistencies. Furthermore, to mitigate redundant data, the mean values of the highly correlated metatarsal sensors (MTK1, MTK3, MTK5) were computed and designated as “MTK values”.

In order to cluster patients according to temperature deviations, key patient-specific metrics were extracted. These comprised the mean and maximum bilateral temperature differences in the metatarsal region (MTK) across the entire SPDF study duration. Subsequently, the K-means clustering method was employed to categorize patients into subgroups based on the temperature-derived metrics^26,27^. K-means clustering is an unsupervised machine learning technique designed to partition N observations into K clusters by assigning each observation to the cluster with the closest mean value, which serves as the cluster prototype. The determination of the optimal number of clusters (K = 2) was achieved through the utilization of the silhouette method^27^. In accordance with the findings of the clustering process, subjects participating in the intervention group were categorized into the following two classifications: cluster 1, characterized by minimal plantar temperature asymmetries; and cluster 2, exhibiting plantar temperature asymmetries.

### Statistics and reproducibility

Categorical variables are reported as frequencies and percentages, and continuous variables as means ± standard deviations (SD) or medians with interquartile ranges (IQR), depending on distribution assessed by the Shapiro–Wilk test. Group differences were assessed using χ² tests with Yates’ continuity correction or Fisher’s exact tests for categorical variables, and independent-sample *t*-tests or Mann–Whitney U tests for continuous variables, as appropriate.

The full follow-up cohort included 239 patients, and analyses of longitudinal sensor-based temperature patterns were restricted to patients with more than 30 days of recordings. The individual patient was the primary statistical unit. Sensor-based temperature recordings were treated as repeated longitudinal measurements within patients and summarized at the patient level to define pre-specified PTDs and bilateral temperature-variability clusters. PIRI symmetry was assessed at the patient level. No independent experimental replicates were performed because this was a post-hoc observational clinical analysis. Reproducibility was supported by pre-specified PTD definitions, unsupervised clustering, and complementary analyses across sensor-based and PIRI-derived data.

The original intervention/control comparison was designed to evaluate diabetic foot outcomes and has been reported previously^19,20^. Because the alert system was not designed as a cardiovascular intervention, intervention allocation was not interpreted as a treatment effect for AF or thromboembolic events in the present follow-up analysis. The primary analyses, therefore, focused on prognostic associations between plantar temperature deviation metrics and subsequent AF and thromboembolic events.

Associations between plantar temperature metrics and AF or thromboembolic outcomes were evaluated using univariate and multivariable logistic regression and Cox proportional hazards models. Multivariable models were adjusted for sex, age (in years), BMI (weight/height^2^), ABI, and duration of diabetes mellitus (in years). Logistic regression was used for the composite outcome and for AF, for which reliable time-of-onset data were unavailable. Cox regression was used for PE, stroke, and PAD, for which time-to-event data were available. Participants with missing diabetes duration (12/118, 10.2%) were excluded from multivariable regression analyses. Statistical significance was defined as a two-tailed *p*-value < 0.05. Analyses were performed in R version 4.2.1, with packages listed in the Supplementary materials.

## Results

### Study participants

In the SPDF study, patients with diabetes and peripheral polyneuropathy were enrolled from January 2018 until March 2021 (Fig. 1a)^19,20^. Participants were randomly assigned to either the control or intervention group in a 1:1 ratio and were observed for the onset of diabetic foot ulcers (Fig. 1b). The intervention group was provided with a telemedical patient monitoring system, utilized for the purpose of daily plantar temperature recordings. This system was equipped with an alert mechanism, designed to trigger in the event of asymmetric temperature elevations. During the course of the study, two participants in the control group died, while 13 participants (six from the control group and seven from the intervention group) withdrew their participation. Moreover, the temperature records of eight participants were insufficient to provide a complete dataset, with a total duration of less than 30 days. Consequently, 260 participants remained eligible for subsequent analyses. The objective of this follow-up study was to evaluate the downshifted plantar foot temperature recordings and to correlate these with the composite of AF and thromboembolic events.

### Follow-up analyses of AF and thromboembolic events

260 participants were contacted via telephone in order to inquire whether they had experienced AF and/or thromboembolic events since the study commencement. A standardized questionnaire was developed to record past medical events of arrhythmias (AF), stroke, PE, and PAD (Fig. 1a). Following the conclusion of the SPDF study, 16 had died, and five were lost to follow-up. Consequently, 239 participants with complete follow-up data were included in the analyses (Fig. 1b).

With regard to all baseline biographical parameters, no statistically significant differences between the control group (*n* = 121) and the intervention group (*n* = 118) were identified (Table 1). The median follow-up duration was 67.1 months (range: 51.7–75.9 months). During the maximum follow-up period, 45 out of 239 participants (18.8%) experienced the composite of AF and thromboembolic events (see Table 1). The prevalence of adverse outcomes was further examined, with arrhythmia observed in 26 participants (10.9%), stroke in six participants (2.5%), PE in four participants (1.7%), and PAD in 11 participants (4.6%). The distribution of AF and thromboembolic events was found to be comparable between the two groups.

**Table 1:** Baseline characteristics and the composite of AF and thromboembolic events of study participants (*n* = 239 patients)

Group	Control (*n* = 121)	Intervention (*n* = 118)	*P*-value	
**Age, years, median (IQR)**	66.0 (11.0)	65.5 (11.0)	0.23	ns
**Sex (female)**	41 (33.9%)	40 (33.9%)	0.99	ns
**Types of diabetes mellitus**			0.44	ns
Type 1	19 (15.7%)	24 (20.3%)		
Type 2	102 (84.3%)	94 (79.7%)		
**Duration of diabetes mellitus, years**^**a**^**, median (IQR)**	16.0 (14.8)	14.5 (16.8)	0.97	ns
**Height, m, mean (SD)**	1.7 (0.1)	1.7 (0.1)	0.48	ns
**Weight, kg, median (IQR)**	91.0 (20.0)	90.0 (24.8)	0.87	ns
**Body mass index (BMI), kg/m**^**2**^**, median (IQR)**	29.7 (5.7)	28.7 (6.5)	0.38	ns
**Max. walking distance without assistances**			0.31	ns
<200	2 (1.7%)	2 (1.7%)		
200─500	10 (8.3%)	4 (3.4%)		
500─1000	12 (9.9%)	8 (6.8%)		
**Neuropathy symptoms score (NSS)**			0.99	ns
Moderate (5–6)	24 (19.8%)	23 (19.5%)		
Severe (7–10)	75 (62.0%)	72 (61.0%)		
**Neuropathy disability score (NDS)**			0.79	ns
Moderate (6–8)	53 (43.8%)	59 (50.0%)		
Severe (9–10)	11 (9.1%)	9 (7.6%)		
**Ankle-brachial index (ABI), right foot**			0.77	ns
≤0.75–0.5	2 (1.7%)	2 (1.7%)		
≤0.9–0.75	10 (8.3%)	7 (5.9%)		
≥1.3	6 (5.0%)	9 (7.6%)		
**Ankle-brachial index (ABI), left foot**			0.38	ns
≤0.75–0.5	1 (0.8%)	4 (3.4%)		
≤0.9–0.75	10 (8.3%)	6 (5.1%)		
≥1.3	5 (4.1%)	7 (5.9%)		
**Foot risk classification**			0.82	ns
Group 2	113 (93.4%)	112 (94.9%)		
Group 3	8 (6.6%)	6 (5.1%)		
**Events**				
Atrial fibrillation (AF, *n* = 26)	13 (10.7%)	13 (11.0%)	0.99	ns
Pulmonary embolism (PE, *n* = 4)	1 (0.8%)	3 (2.5%)	0.37	ns
Stroke (*n* = 6)	3 (2.5%)	3 (2.5%)	0.99	ns
Peripheral artery disease (PAD, *n* = 11)	5 (4.1%)	6 (5.1%)	0.97	ns
**Overall (n** = **45)**	22 (18.2%)	23 (19.5%)	0.93	ns

Categorical variables are presented as *n* (%), and continuous variables are described as mean ± SD or median [IQR]. Group comparisons were performed using chi-squared tests or Fisher’s exact tests for categorical variables and *t*-tests or Mann–Whitney U tests for continuous variables, according to their distribution. All *p*-values are two-sided, and no adjustment was made for multiple comparisons. Bold values indicate two-sided *p* < 0.05.

*ABI* ankle-brachial index, *AF* atrial fibrillation, *BMI* body mass index, *IQR* interquartile range, *NDS* neuropathy disability score, *NSS* neuropathy symptoms score, *PAD* peripheral artery disease, *PE* pulmonary embolism, *SD* standard deviation.

^a^Diabetes duration is only available from 102 patients in the control group and 106 patients in the intervention group.

To interrogate potential links between plantar temperature characteristics and incident AF and/or thromboembolic events, we implemented three complementary analytic strategies. (i) Sensor-derived plantar temperature time series were adjudicated for plantar temperature downshifts (PTDs) and dichotomized as present versus absent. (ii) The same time series underwent unsupervised clustering to characterize inter-foot temperature asymmetry, classified as yes versus no. (iii) Plantar infrared thermograms were independently classified as symmetric (normal) versus asymmetric (Fig. 1c). Risk strata derived from each method were then evaluated for associations with AF and/or thromboembolic outcomes. Results are presented in the subsequent sections. Time-to-event differences across PTD status, asymmetry clustering, and PIRI asymmetry are additionally summarized using Kaplan–Meier curves (Supplementary Fig. 1).

### Post-hoc analysis of plantar temperature recordings

**Fig. 2:**
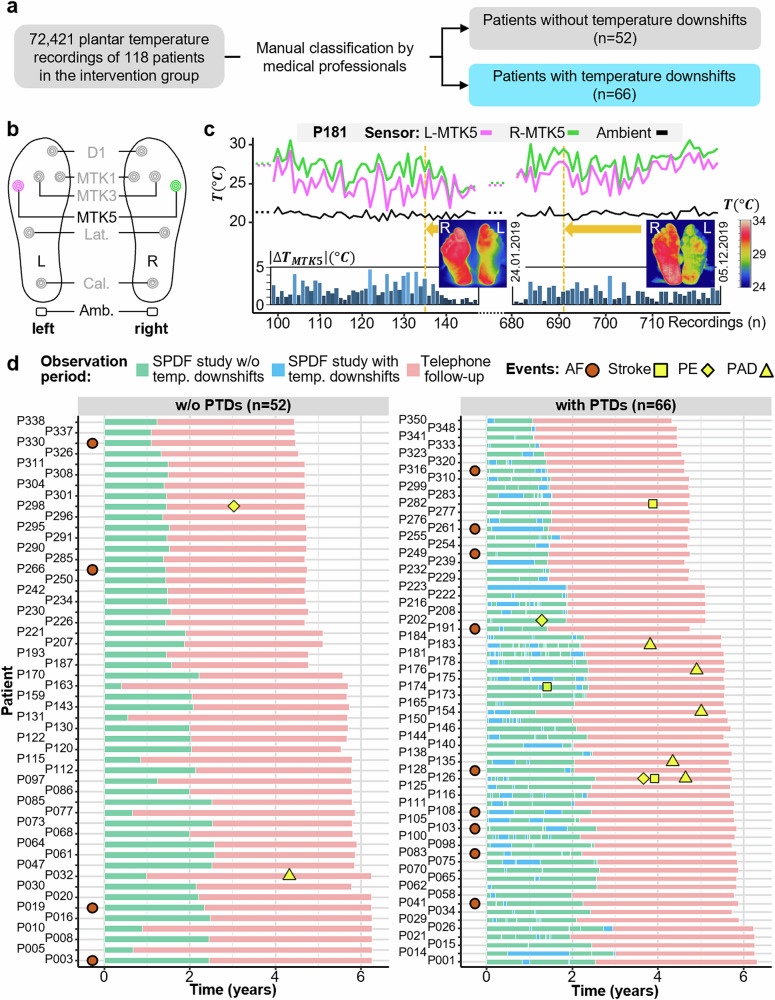
Post-hoc analyses of plantar temperature recordings (*n* = 118 patients). **a** Post-hoc analyses of plantar temperature recordings resulted in the classification of two subgroups of participants: 66 with and 52 without PTDs. **b** Overview of plantar temperature sensor positions and bilateral symmetry mapping. **c** Example of temperature recordings from a representative participant who presented with repeated episodes of PTDs in the left forefoot area (sensor L-MTK5). Two infrared images are presented with corresponding temperature downshifts in the left lateral forefoot. **d** Visualization of the composite outcome (AF and thromboembolic events) by PTD status. AF atrial fibrillation, MTK metatarsal, PAD peripheral arterial disease, PE pulmonary embolism, PTDs transient plantar temperature downshifts, T absolute plantar foot temperatures (°C), ∆T left–right temperature difference at the same sensor site (e.g., left MTK3 minus right MTK3).

A post-hoc analysis of plantar temperature sensor recordings was conducted under the supervision of three medical professionals (a study coordinator and two physicians supervising the SPDF study)^19,20^. This analysis was carried out in conjunction with records on daily foot assessment by the patient, plantar and dorsal foot photographs, and infrared foot imaging (see Fig. 2a). The analysis incorporated a total of 72,421 temperature recordings obtained from 118 patients in the intervention group. The plantar temperature values recorded by five sensor pairs (MTK1, MTK3, MTK5, lateral, and calcaneus, Fig. 2b) were normalized by subtracting the ambient temperature values. The latter were recorded by additional sensors integrated into Bluetooth devices. Subsequently, temperature variations and asymmetries over time were analyzed in conjunction with infrared imaging (Supplementary Figs. 2−5) and patient-reported daily foot assessments. An example of a patient (P181) with an episode of plantar temperature downshift in the left forefoot area (sensor L-MTK5) associated with repeated transient ischemia, as visualized by infrared imaging, is shown in Fig. 2c. Subsequent post-hoc analyses of 118 participants in the intervention group subdivided them into two categories: those without plantar temperature downshifts (PTD, *n* = 52, 44%) and those with such downshifts (*n* = 66, 56%; Fig. 2a, d). In the subgroup with PTDs, AF and/or thromboembolic events were observed in 17 subjects (25.8%). Conversely, the incidence of events was *n* = 6 (11.5%) in patients without PTDs. However, this trend did not reach a level of statistical significance (*p* = 0.09, Table 2) and should be interpreted as exploratory. Consistent time-to-event visualization is provided in the Kaplan–Meier analysis for PTD strata (Supplementary Fig. 1a; log-rank *p* = 0.12). The events documented for the two subgroups are illustrated in Fig. 2d at the level of individual patients. It is important to note that some patients exhibited repeated episodes of PTDs, as indicated by blue coloring in Fig. 2d and white stars within the IR images (Supplementary Figs. 2−5).

**Table 2:** Summary of AF and thromboembolic events by patient subgroups in the intervention group

Analysis method	Post-hoc analysis of the temperature sensor recordings			Unsupervised clustering of the temperature sensor recordings			Classification of infrared thermography		
Group (*n* = 118)	w/o PTDs (*n* = 52)	with PTDs (*n* = 66)	*P*-value	Cluster 1 (*n* = 71)	Cluster 2 (*n* = 47)	*P*-value	Normal (*n* = 81)	Asymmetric (*n* = 37)	*P*-value
**Follow-up** **in months**	67.4 (13.1)	66.7 (12.5)	0.86	66.5 (12.9)	66.8 (12.5)	0.40	66.4 (12.9)	68.0 (12.6)	0.24
**AF (n** = **13)**	4 (7.7%)	9 (13.6%)	0.47	5 (7.0%)	8 (17.0%)	0.16	4 (4.9%)	9 (24.3%)	**0.003**
**PE (n** = **3)**	1 (1.9%)	2 (3.0%)	0.99	1 (1.4%)	2 (4.3%)	0.56	0 (0%)	3 (8.1%)	**0.029**
**Stroke (n** = **3)**	0	3 (4.5%)	0.25	2 (2.8%)	1 (2.1%)	0.99	0 (0%)	3 (8.1%)	**0.029**
**PAD (n** = **6)**	1 (1.9%)	5 (7.6%)	0.23	1 (1.4%)	5 (10.6%)	**0.04**	1 (1.2%)	5 (13.5%)	**0.011**
**Overall (n** = **23)**	6 (11.5%)	17 (25.8%)	0.09	7 (9.9%)	16 (34.0%)	**0.003**	5 (6.2%)	18 (48.6%)	**<.0001**

Data are shown for patients in the intervention group with available subgroup classifications (*n* = 118 patients). The value of *n* refers to individual patients unless otherwise stated. A post-hoc analysis of temperature recordings was performed by the examiners and medical supervisors of the SPDF study and complemented by unsupervised clustering of temperature recordings. Infrared thermography was classified by three independent examiners who were unaware of PTD status. Data are presented as counts (*n*) and percentages (%), means with standard deviations (SD), or medians with interquartile ranges (IQR), as appropriate. Group comparisons were performed using chi-squared tests or Fisher’s exact tests for categorical variables and independent-sample *t*-tests or Mann–Whitney U tests for continuous variables, according to data distribution. All *p*-values are two-sided, and no adjustment was made for multiple comparisons. Exact *p*-values are reported where available. Bold values indicate two-sided *p* < 0.05.

*AF* atrial fibrillation, *IQR* interquartile range, *PAD* peripheral artery disease, *PE* pulmonary embolism, *PTDs* plantar temperature downshifts.

### Clustering analysis of plantar temperature characteristics

**Fig. 3:**
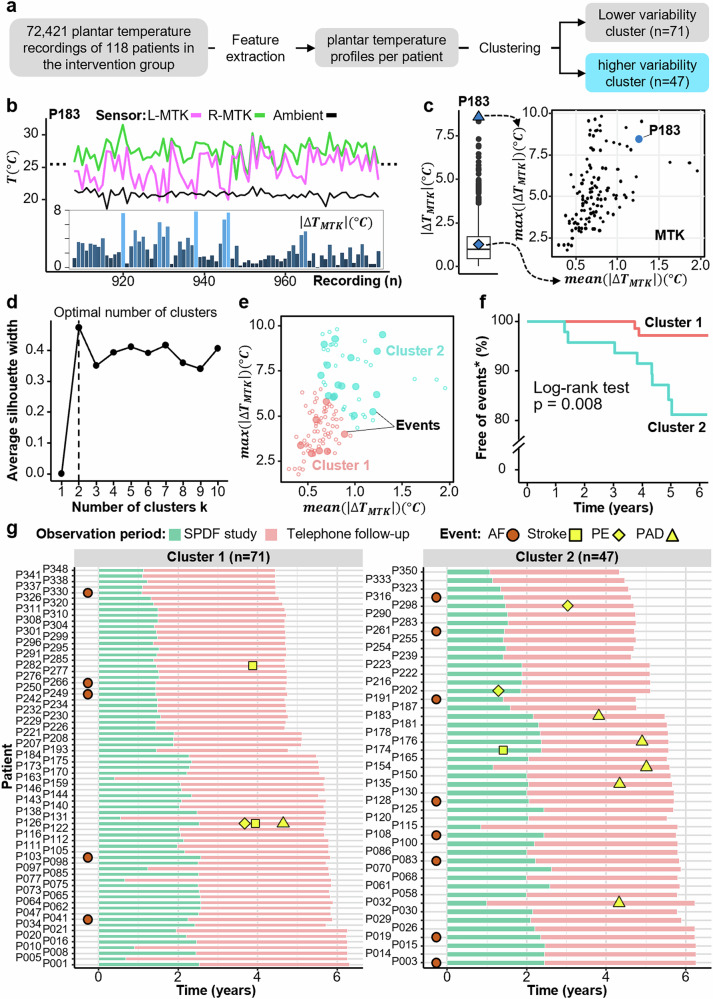
Clustering analyses of plantar temperature recordings (*n* = 118 patients). **a** Unsupervised clustering classified participants into two temperature-variability clusters (lower- vs higher-variability) based on plantar temperature patterns. **b** Representative recordings from participant P183 showing repeated plantar temperature asymmetries, with between-foot differences reaching up to 8 °C in the metatarsal region. **c** Metric extraction for clustering, including maximum and mean left–right temperature asymmetry in the metatarsal (MTK) region; P183 is highlighted in blue. **d** Selection of the number of clusters using the silhouette method. **e** Distribution of the composite outcome (AF and thromboembolic events) across clusters (symbol fill indicates participants with events). **f** Kaplan–Meier curves for time-to-event outcomes (stroke, PAD, and PE) by cluster. The *p*-value was obtained from a two-sided log-rank test; no adjustment was made for multiple comparisons. **g** Visualization of ascertained outcomes within clusters over follow-up. AF atrial fibrillation, MTK metatarsal, PAD peripheral arterial disease, PE pulmonary embolism, T absolute plantar temperature (°C), ΔT left–right temperature difference at the same sensor site (e.g., left MTK3 minus right MTK3).

To further explore the plantar temperature profiles in relation to incident outcomes, an unsupervised clustering analysis was performed. The objective was to categorize participants into subgroups based on their thromboembolic risk levels (Fig. 3a). The previously mentioned process of temperature metric extraction was performed with the entire set of 72,421 temperature recordings from 118 patients. The mean and maximum bilateral temperature differences for the metatarsal area (MTK) in each participant were calculated, as illustrated in Fig. 3b, c. Cluster analysis using these two key temperature metrics resulted in two distinct patient subgroups: cluster 1 (*n* = 71) and cluster 2 (*n* = 47; Fig. 3d). Cluster 1 exhibited minimal plantar temperature asymmetries, while cluster 2 exhibited significantly greater asymmetries, as evidenced by the following differences: maximum bilateral temperature differences of 4.1 ± 1.1 °C in cluster 1 vs. 7.1 ± 1.6 °C (*p* < 0.0001) in cluster 2 and mean bilateral temperature differences of 0.6 ± 0.1 °C in cluster 1 vs. 1.0 ± 0.3 °C in cluster 2 (*p* < 0.0001) (Fig. 3e). The findings indicate a marked deviation in plantar thermoregulation among patients in cluster 2.

AF and/or thromboembolic events were significantly more prevalent in cluster 2, which was hereafter referred to as the higher-variability cluster. This cluster exhibited 16 out of 47 patients experiencing events (34%), in contrast to the 7 out of 71 patients (9.9%) observed in cluster 1, which was labeled the lower-variability cluster (*p* = 0.003; Table 2). In comparison with the lower-variability cluster, the OR for incident AF and/or thromboembolic events was 4.72 (95% CI: 1.82–13.39; *p* = 0.002) in the higher-variability cluster 2 (see Table 3). Following adjustment for confounding variables, including sex, age, BMI, and diabetes duration, patients in cluster 2 still exhibited a significantly higher risk of new onset AF and/or thromboembolic events (adjusted OR: 13.03; 95% CI: 3.82–53.90; *p* < 0.001). The robustness of this association was maintained upon additional adjustment for the covariate ABI (adjusted OR: 13.51; 95% CI: 3.85–58.61; *p* < 0.001), as demonstrated in Table 3.

**Table 3:** Logistic regression analysis for the association between obtained plantar temperature-variability clusters and the composite of AF and thromboembolic events (stroke, PE, PAD)

Model	Variable	OR	95% CI	*P*-value
1	Cluster 2 vs 1	4.72	1.82–13.39	0.002
**2**^**a**^	Age (per year)	1.04	0.97–1.12	0.279
	Sex (Male vs Female)	3.91	1.08–17.46	0.051
	BMI (kg/m^2^)	0.90	0.79–0.99	0.064
	Diabetes duration (per year)	0.97	0.91–1.01	0.205
	**Cluster 2 versus 1**	**13.03**	**3.82–53.90**	**<0.001**
**3**^**a**^	Age (per year)	1.04	0.98–1.10	0.206
	Sex (Male vs Female)	2.00	0.63–7.71	0.268
	BMI (kg/m^2^)	0.96	0.87–1.04	0.394
	Diabetes duration (per year)	0.97	0.92–1.02	0.266
	**ABI (right)**	**50.32**	**0.11–35,857**	**0.207**
	**ABI (left)**	**0.01**	**<0.001–5.39**	**0.154**
**4**^**a**^	Age (per year)	1.04	0.97–1.12	0.290
	Sex (Male vs Female)	3.56	0.96–16.12	0.073
	BMI (kg/m^2^)	0.90	0.79–0.99	0.063
	Diabetes duration (per year)	0.97	0.91–1.02	0.249
	ABI (right)	66.90	0.09–198,279	0.250
	ABI (left)	0.01	<0.001–9.09	0.210
	**Cluster 2 versus 1**	**13.51**	**3.85–58.61**	**<0.001**

Data are shown for *n* = 118 patients in Model 1 and *n* = 106 patients in Models 2–4 after exclusion of 12 patients with missing diabetes duration; *n* refers to individual patients. Model 1 was unadjusted. Model 2 was adjusted for age, sex, BMI, and diabetes duration. Model 3 was constructed to evaluate the independent prognostic value of ABI and was adjusted for age, sex, BMI, and diabetes duration. Model 4 was fully adjusted and included ABI, separately for the right and left side, and the temperature-variability cluster. ORs are reported with 95% CIs. *P*-values are two-sided and were derived from logistic regression models. No adjustment was made for multiple comparisons. Exact *p*-values are reported where available. Bold values indicate two-sided *p* < 0.05.

*ABI* ankle-brachial index, *AF* atrial fibrillation, *BMI* body mass index, *CI* confidence interval, *OR* odds ratio, *PAD* peripheral artery disease, *PE* pulmonary embolism.

^a^Twelve patients with missing data on diabetes duration were excluded from models 2–4.

For time-to-event outcomes (PE, stroke, PAD), the same analysis demonstrated significantly increased risk for thromboembolic outcomes in cluster 2 across all adjustment levels, including ABI (adjusted HR: 13.31; 95% CI: 1.47–120.39; *p* = 0.021; Table 4). The distribution of events across clusters is visualized in Fig. 3e, the Kaplan–Meier analysis of time-to-event outcomes between the obtained clusters is visualized in Fig. 3f (log-rank *p* = 0.008). The temporal occurrence and dissemination of AF and thromboembolic events within the two patient clusters are depicted in Fig. 3g.

**Table 4:** Cox proportional hazards regression analysis for the association between obtained plantar temperature-variability clusters and thromboembolic time-to-event outcomes (stroke, PE, PAD)

Model	Variable	HR	95% CI	*P*-value
1	Cluster 2 vs 1	6.23	1.32–29.4	0.021
**2**^**a**^	Age (per year)	0.99	0.91–1.09	0.930
	Sex (Male vs Female)	2.07	0.40–10.64	0.384
	BMI (kg/m^2^)	0.92	0.80–1.06	0.255
	Diabetes duration (per year)	0.98	0.92–1.05	0.538
	**Cluster 2 versus 1**	**14.79**	**1.74–125.63**	**0.014**
**3**^**a**^	Age (per year)	0.99	0.92–1.06	0.727
	Sex (Male vs Female)	1.32	0.24–7.32	0.752
	BMI (kg/m^2^)	0.96	0.85–1.08	0.529
	Diabetes duration (per year)	0.98	0.92–1.05	0.653
	**ABI (right)**	**0.61**	**<0.001–11552.11**	**0.921**
	**ABI (left)**	**0.02**	**<0.001–121.30**	**0.362**
**4**^**a**^	Age (per year)	0.97	0.89–1.06	0.489
	Sex (Male vs Female)	1.91	0.34–10.63	0.458
	BMI (kg/m^2^)	0.93	0.81–1.06	0.273
	Diabetes duration (per year)	0.99	0.92–1.06	0.728
	ABI (right)	0.67	<0.001–1949.3	0.922
	ABI (left)	0.05	<0.001–69.36	0.418
	**Cluster 2 versus 1**	**13.31**	**1.47–120.39**	**0.021**

Data are shown for *n* = 118 patients in Model 1 and *n* = 106 patients in Models 2–4 after exclusion of 12 patients with missing diabetes duration; *n* refers to individual patients. Model 1 was unadjusted. Model 2 was adjusted for age, sex, BMI, and diabetes duration. Model 3 was constructed to evaluate the independent prognostic value of ABI and was adjusted for age, sex, BMI, and diabetes duration. Model 4 was fully adjusted and included ABI, separately for the right and left side, and the temperature-variability cluster. HRs are reported with 95% CIs. *P*-values are two-sided and were derived from Cox proportional hazards regression models. No adjustment was made for multiple comparisons. Exact *p*-values are reported where available. Bold values indicate two-sided *p* < 0.05.

*ABI* ankle-brachial index, *BMI* body mass index, *CI* confidence interval, *HR* hazard ratio, *PAD* peripheral artery disease, *PE* pulmonary embolism.

^a^Twelve patients with missing data on diabetes duration were excluded from models 2–4.

### Classification of plantar infrared thermography

**Fig. 4:**
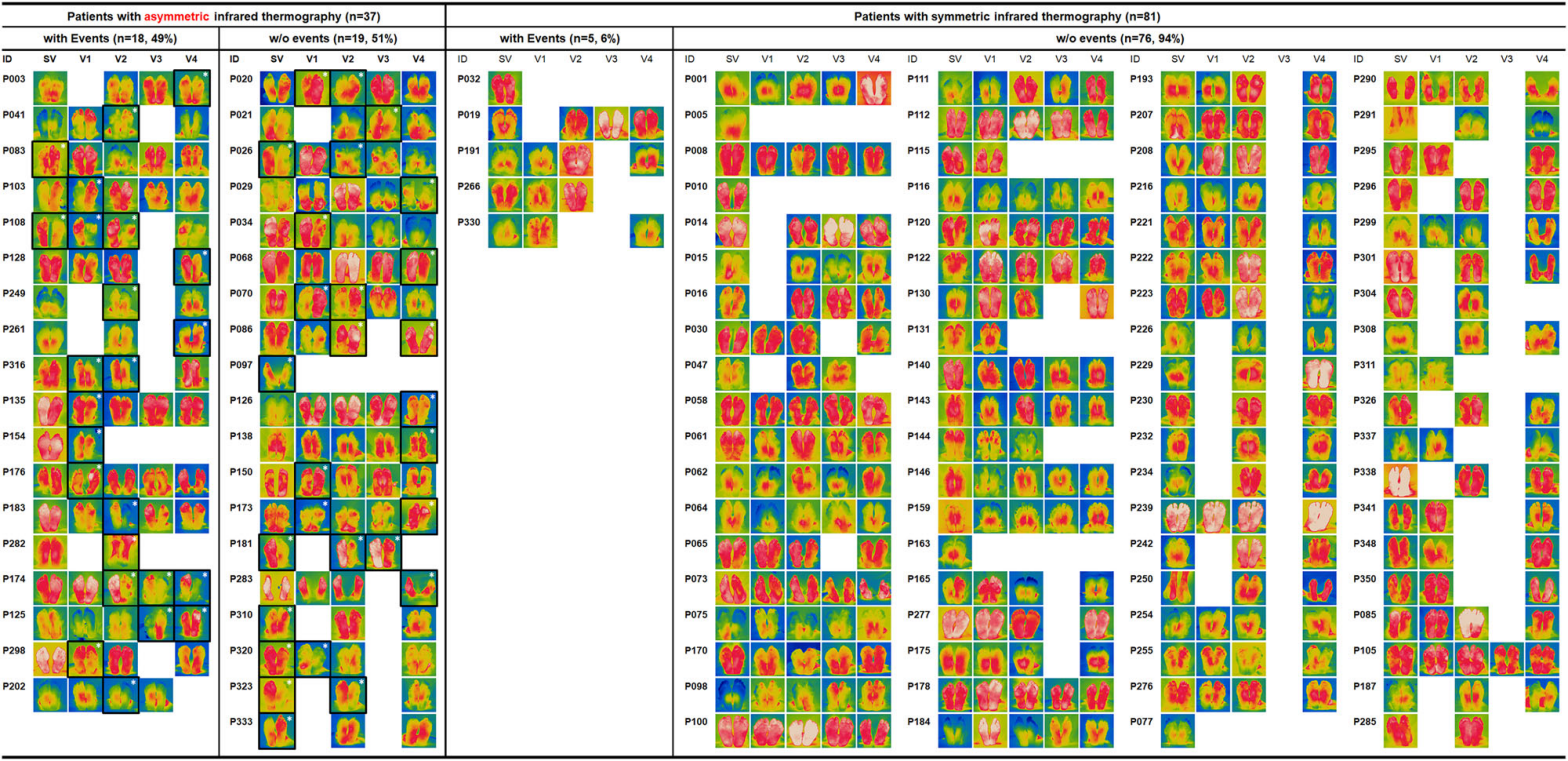
Representative plantar infrared imaging (PIRI) from the intervention group (*n* = 118 patients). Patients with asymmetric plantar temperature patterns are highlighted with black rectangles and white stars (37/118, 31%). During follow-up, 23/118 experienced the composite of AF and thromboembolic events (stroke, PE, PAD). The composite endpoint occurred more frequently in patients with PIRI asymmetry than in those with symmetric patterns: 18/37 (48.6%) vs 5/81 (6.2%), *p* < 0.0001. The *p*-value was obtained using a two-sided Fisher’s exact test. No adjustment was made for multiple comparisons. AF atrial fibrillation, PAD peripheral arterial disease, PE pulmonary embolism.

Overall, 1145 PIRIs were available from all participants (Supplementary Figs. 2−5). Asymmetric temperature distributions were observed in 68/239 (28.5%) individuals with 31/121 (25.6%) in the control and 37/118 (31.3%) in the intervention group (Table 2, Supplementary Table 1, Fig. 4). Patients with PIRIs asymmetries reached the composite endpoint of AF and/or thromboembolic events significantly more often than those with symmetric temperatures visualized by PIRIs, both in the control (48.4% vs 7.7%; *p* < 0.0001; Supplementary Table 1) as well as in the intervention groups (48.6% vs 6.2%; *p* < 0.0001; Table 2). Kaplan–Meier curves for event-free survival by PIRI asymmetry status are shown in Supplementary Fig. 1c (log-rank *p* < 0.0001). In the control group, temperature asymmetries were associated with AF (25.8% vs 5.5%; *p* = 0.004) and PAD (12.9% vs 1.1%; *p* = 0.015), whereas differences for PE (3.2% vs 0%; *p* = 0.256) and stroke (6.4% vs 1.1%; *p* = 0.161) were not statistically significant due to low event numbers (Supplementary Figs. 4 and 5). In the intervention group, asymmetries were associated with higher rates of AF (24.3% vs 4.9%; *p* = 0.003), PE (8.1% vs 0%; *p* = 0.029), stroke (8.1% vs 0%; *p* = 0.029), and PAD (13.5% vs 1.2%; *p* = 0.011) (Supplementary Figs. 2 and 3). The plantar infrared imaging and temperature sensor findings were overall concordant. Asymmetric thermography was prevalent in participants with PTD (84% vs. 16%, *p* < 0.001), similarly was the prevalence in the higher-variability cluster (65% vs. 35%, *p* < 0.001; Supplementary Table 2).

## Discussion

For this study, we introduce the label “plantar temperature downshift” (PTD) to describe a phenomenon that became apparent within the SPDF study. It was assigned in patients with (i) similar values for plantar and ambient temperatures, (ii) asymmetries (calculations of temperature differences between the feet reached values up to 5.9 °C), (iii) temperature downshifts persisting for a minimum of 36 h, and (iv) clinical evaluation to exclude the possibility of artificial downshifts (e.g., wound plaster or other forms of coverage).

In the intervention group of the SPDF study with recordings of more than 30 days, the detection of PTDs in 66/118 patients attracted our attention and prompted this observational post-hoc follow-up study to scrutinize underlying mechanisms and causes. The hypothesis was formulated that the occurrence of PTDs may be part of a systemic state of activated coagulation, thrombus formation, and cardiovascular thromboembolic events. The primary focus of the present study was the identification of AF and thromboembolic risk constellations. This was based on the assumption that such diseases may have incited clinically apparent symptoms that may be amenable to the interviewer in a standardized telephone questionnaire. The following endpoints were defined: history of AF, acute cerebral stroke, and clinically apparent peripheral artery disease (PAD; classified as Fontaine stages IIb or higher^28^), and pulmonary embolism (PE). All participants were diagnosed with long-standing diabetes and peripheral neuropathy (NSS ≥ 5: 81% [194/239], NDS ≥ 6: 55% [132/239]). It is noteworthy that none of the patients were aware of this condition, a likely consequence of impaired or absent temperature discrimination and pathological Tiptherm findings. All participants were examined for peripheral artery disease, with the vast majority exhibiting an ABI score of more than 0.75 (233/239, 97%), and no impairment in walking distance below 2500 m. Control ABI determinations revealed that these were lower at follow-up examinations in 35/66 patients with PTDs.

The comparative analyses comprised three subgroups: patients in the control group without any data on plantar temperatures (*n* = 121), patients in the intervention group with regular temperature recordings without PTDs (*n* = 52), and those with PTDs (*n* = 66). The follow-up period lasted for a median of 67.1 months (range 51.7 to 75.9 months). The event analysis revealed that the rate of CV endpoints and AF was similar between the intervention and control groups (19.5% versus 18.2%, *p* = 0.93; see Table 1 for details). This rate is commensurate with others reported for patients with a high-risk profile who have been diagnosed with diabetes^29^.

A significant aspect of AF is the heightened probability of cerebral and systemic embolic events, predominantly of an ischemic nature, though not of a hemorrhagic nature^30,31^. For example, the ATRIA study showed that the duration of diabetes > three years compared to that of less than three years was associated with an increased risk of ischemic stroke (adjusted HR 1.74)^32^.

The primary analyses conducted on PTDs relied on manual data aggregation and interpretation, with ambient temperature values serving as the reference point. The second approach, which involved the calculation of mean and maximal temperature deviations within the forefoot, enabled the clustering of patients. The latter approach successfully distinguished patients at high risk of the composite of AF and thromboembolic events (*p* = 0.003). The enhanced efficacy of the second approach may be attributable to two potential factors. Firstly, the presence of subtle variations in PTDs when only a subarea of the foot is subject to ischemia. Secondly, incomplete occlusions of peripheral vessels result in plantar temperatures that may persist above the ambient temperature reference.

A significant challenge in the study of PTDs is the absence of a formalized clinical definition of this condition. The prevailing literature focuses primarily on unilateral plantar temperature elevations as early warning signs for foot ulceration. In contrast, to the best of our knowledge, there has not been a systematic investigation of unilateral hypothermia (under whatever condition and related to clinical risk constellations).

We used multivariable models that included established AF and thromboembolic risk factors (age, sex, BMI, diabetes duration, and ABI) to evaluate whether clustering based on plantar temperature values could predict the composite of AF and thromboembolic outcomes independently. We incorporated ABI as a surrogate for peripheral arterial status, given its clinical relevance in thromboembolic risk stratification. However, ABI (right and left) showed limited predictive value in our models, being associated with notably wide confidence intervals (see Tables 3 and 4), likely due to low variability and limited event counts. In contrast, the association of cluster 2 with the composite of AF and thromboembolic events remained consistently strong and statistically significant across all models (see Tables 3 and 4). In the fully adjusted Cox regression model, the hazard ratio for cluster 2 was 13.3 (95% CI: 1.4–120.4), and in the logistic regression model, the OR was 13.5 (95% CI: 3.8–58.6). These results suggest that plantar temperature asymmetries captured by clustering analysis may reflect underlying subclinical thromboembolic processes that are not fully captured by traditional indicators such as ABI. A more detailed analysis of the infrared images taken at different study visits corroborated the hypothesis of thromboembolic events, with regions exhibiting reduced temperature values being delineated by color changes associated with angiosomes (e.g., calcaneus, forefoot; see Supplementary Figs. 2–5) or the entire foot. Visualization of the impaired blood supply of a whole foot was seen in four patients (P103, P108, P183, and P174).

The present study has several limitations: A notable limitation of the present study is the absence of medical records from each patient, which were not obtained through contact with attending physicians. It is possible that patients lack awareness of AF and thromboembolic diseases, which could have resulted in the provision of inconclusive information. It is evident that the coronary artery status or myocardial infarction of the majority of patients with diabetes is not subject to question. Most of these patients with advanced age suffer from accelerated coronary artery disease, and a systematic diagnostic workup on the coronary status is not available and not the focus of this study. Without systematic testing and recording by a specialist, the quality of such data would be rather imprecise. We therefore concluded that this would be beyond the scope of a telephone questionnaire. Similarly, our post-hoc analyses are not based on clinical examinations of patients by a physician. No test results obtained by electrocardiogram (ECG), echocardiography, or angiography of the lower limbs were included, which is a major weakness of the data quality. The data are based solely on self-reporting by participating patients, and the study cohort is too small for an endpoint analysis. Furthermore, our patients are subject to selection bias given their inclusion in our SPDF study protocol. The inclusion criteria were quite strict with regard to the presence of peripheral neuropathy and the absence of macroangiopathy. Importantly, the mobile phone–based alert system should be interpreted as part of the original diabetic foot prevention trial rather than as an intervention intended to modify cardiovascular risk^19,20^. The present findings therefore support the prognostic relevance of plantar temperature deviation metrics, rather than a causal effect of the alert system on AF or thromboembolic events.

The generalizability of our findings is limited by the highly specific inclusion criteria of the SPDF study. All participants had diabetes and moderate-to-severe peripheral neuropathy, and most had a preserved ABI (>0.75). Patients with macroangiopathy, critical limb ischemia, or active foot ulcers were excluded. Consequently, our cohort may not be representative of the wider diabetic population. Additionally, the temperature-monitoring intervention required daily adherence and a degree of digital literacy, which may have resulted in participation being biased toward individuals who are more health-conscious or mobile. These constraints should be considered when interpreting and applying our findings in other clinical contexts. Our findings must be reproduced by independent cohorts and predefined endpoint studies before they can be generalized.

While the PTDs are suggestive of thromboembolic events, direct proof of such events has not been obtained, for example, through digital subtraction angiography. In one patient, an episode of acute myocardial infarction occurred at the same time as the PTD onset. It is notable that there are potential positive effects of transient limb ischemia episodes, which may be regarded as preconditioning. Ischemic preconditioning reduces local tissue injury caused by subsequent ischemia-reperfusion (IR) and also has beneficial effects on IR injury in tissues distant from those undergoing preconditioning^33^. Such effects were not considered in this small cohort.

The strengths of our analyses include the long-term setup, a thorough repeated examination of all patients over this long period, highly precise determinations of plantar temperatures at pre-selected sites, and a large number of individual temperature recordings, which allow data to be interpreted in an unprecedented way.

In routine care, plantar thermometry could serve as a low-burden home-monitoring procedure that may trigger an alarm in all cases with repeated PTDs. The relevant information needs to be confirmed by medical caregivers. Confirmatory examination results, such as abnormalities within ECGs or findings by duplex ultrasound, will be sought. Ultimately, the risk constellation may be ameliorated by prescribing anticoagulants.

Future work should prospectively validate thermometry-derived data in independent cohorts with pre-specified PTD definitions and a reproducible clustering pipeline, using adjudicated outcomes from medical records/registries. Validation should assess cluster stability and incremental prognostic value beyond established clinical predictors. Ultimately, pragmatic studies are needed to test whether thermometry-triggered clinical pathways (e.g., AF screening and targeted prevention strategies) improve patient-relevant outcomes.

In conclusion, our data indicate that plantar temperature monitoring and repeated PIRI were associated with a higher incidence of AF and thromboembolic events in this high-risk cohort, and may help inform hypothesis-generating risk stratification. Clustering analysis of bilateral temperature variability can further refine risk stratification. If prospectively validated, these approaches have the potential to improve patient care and AF and thromboembolic risk classification. Prospective studies with objective evaluations are warranted, including ECG monitoring, arrhythmia recording systems, and evaluation of targeted anticoagulation in patients with PTDs.

## Supplementary information

Supplementary Information

Description of Additional Supplementary Files

Supplementary Data 1

## Data Availability

The source data underlying the graphs, charts, and plotted data presented in Figs. 1–4 are provided with this article as Supplementary Data 1. The data analyzed in this study include clinical baseline data, sensor-based plantar temperature recordings, plantar infrared imaging data, and follow-up information on atrial fibrillation and thromboembolic events. These data are stored on secure institutional servers at the Otto-von-Guericke University Hospital Magdeburg. De-identified data relevant to the findings are available from the corresponding author upon reasonable request. Requests will be reviewed within 8 weeks, and access may be granted for scientifically justified purposes, subject to institutional approval, applicable data protection regulations, and a data use agreement where required. Public deposition of the full raw dataset is restricted because the dataset contains sensitive patient-level clinical and imaging data. No public accession codes or dataset DOIs are available for the restricted raw dataset. The data will be available at least for 10 years from the publication date.

## Code availability

The analysis code used in this study is available at https://github.com/AntaoDevRep/SPDF-Follow-up-Study and has been archived on Zenodo under the version-specific 10.5281/zenodo.20710374^34^.
