# Supplementary material for: Asymmetric plantar temperature downshifts are associated with atrial fibrillation and thromboembolic events: an observational post hoc analysis of the SmartPreventDiabeticFeet study
Source: Commun Med (Lond). 2026 Jul 31;6:424. doi: 10.1038/s43856-026-01811-3 (PMC13427832; doi:10.1038/s43856-026-01811-3)
Supplement: Supplementary file 1 — Supplementary Information [file 43856_2026_1811_MOESM1_ESM.pdf]

Supplementary Information

This appendix has been provided by the authors to give readers additional information about their work.

Supplement to: Ming A, et al. Asymmetric plantar temperature downshifts are associated with atrial fibrillation and thromboembolic events: an observational post hoc analysis of the SmartPreventDiabeticFeet study

(PDF updated 22.06.2026)

Table of Contents

1 Supplementary tables 2

1.1 Suppl. Table 1. Summary of atrial fibrillation and thromboembolic events by infrared thermography pattern in the control group. 2

1.2 Suppl. Table 2. Concordance between infrared thermography and sensor-derived plantar temperature recordings in the intervention group (n = 118). 3

2 Supplementary tigures 4

2.1 Suppl. Figure 1. Kaplan–Meier analyses by plantar thermometry indicators. 4

2.2 Suppl. Figure 2. Infrared thermography in intervention participants with atrial fibrillation and thromboembolic events (n = 23). 5

2.3 Suppl. Figure 3. Infrared thermography in intervention participants without atrial fibrillation and thromboembolic events (n = 95). 7

2.4 Suppl. Figure 4. Infrared thermography in control participants with atrial fibrillation and thromboembolic events (n = 22). 15

2.5 Suppl. Figure 5. Infrared thermography in control participants without atrial fibrillation and thromboembolic events (n = 99). 17

2.6 Suppl. Figure 6. Preprocessing of plantar temperature recordings. 25

3 Smart Prevent Diabetic Feet Study protocol 26

4 STROBE checklist 27

5 R package environment 29

1 Supplementary tables

1.1 Suppl. Table 1. Summary of atrial fibrillation and thromboembolic events by infrared thermography pattern in the control group.

Group	Control (n=121)		
	normal (n=90)	asymmetric (n=31)	P value
**AF (n=13)**	5 (5.5%)	8 (25.8%)	**0.004**
**PE (n=1)**	0	1 (3.2%)	0.256
**Stroke (n=3)**	1 (1.1%)	2 (6.4%)	0.161
**PAD (n=5)**	1 (1.1%)	4 (12.9%)	**0.015**
**Overall (n=22)**	7 (7.7%)	15 (48.4%)	**<.0001**

Data are presented as counts (n) and percentages (%). Group comparisons were performed using Chi-square tests or Fisher's exact test. All tests were two-sided, with $p < 0.05$ considered statistically significant. Bold values indicate significance. Overall refers to the composite endpoint of AF, PE, stroke, or PAD. AF: atrial fibrillation; PAD: peripheral artery disease; PE: pulmonary embolism.

1.2 Suppl. Table 2. Concordance between infrared thermography and sensor-derived plantar temperature recordings in the intervention group (n = 118).

Subgroups of the intervention group (n=118)		Classification of infrared thermography		P value
		normal (n=81)	asymmetric (n=37)	
Post-hoc analysis of plantar temperature recordings	w/o PTDs (n=52)	46 (57%)	6 (16%)	<0.0001
	with PTDs (n=66)	35 (43%)	31 (84%)	
Clustering analysis of plantar temperature characteristics	cluster 1 (low-risk, n=71)	58 (72%)	13 (35%)	0.0004
	cluster 2 (high-risk, n=47)	23 (28%)	24 (65%)	

Infrared thermography findings (normal vs. asymmetric) were compared with sensor-derived plantar temperature recordings, including post-hoc PTD classification and clustering analysis of plantar temperature characteristics. Asymmetric thermography was significantly more prevalent in participants with PTD compared with those without PTD (84% vs. 16%, $p < 0.001$), and in the high-risk cluster compared with the low-risk cluster (65% vs. 35%, $p < 0.001$), indicating overall concordance between the two measurement approaches. PTDs: transient plantar temperature downshifts.

2 Supplementary tigures

2.1 Suppl. Figure 1. Kaplan–Meier analyses by plantar thermometry indicators.

Kaplan–Meier curves showing event-free survival over follow-up, stratified by (a) presence vs absence of plantar temperature downshifts (PTDs) from sensor-equipped insole recordings, (b) unsupervised clusters of bilateral plantar temperature asymmetry (C1 vs C2) derived from asymmetry metrics, and (c) plantar infrared imaging (PIRI) asymmetry (yes vs no). The endpoint is the composite of atrial fibrillation and thromboembolic events. P values are from log-rank tests. Numbers at risk are shown below each panel.

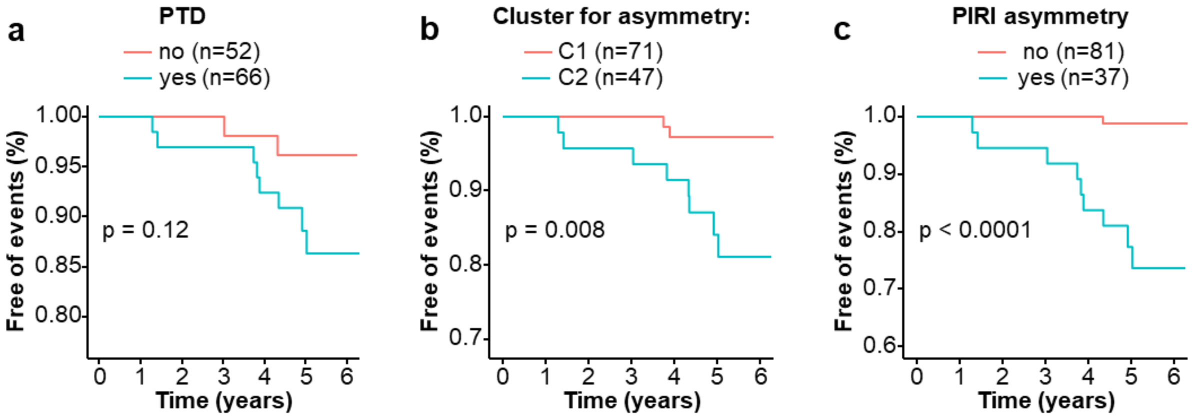

2.2 Suppl. Figure 2. Infrared thermography in intervention participants with atrial fibrillation and thromboembolic events (n = 23).

Eighteen of 23 participants (78%) exhibited asymmetric infrared patterns; corresponding images are marked with a white star. Events include atrial fibrillation (AF), stroke, pulmonary embolism (PE), and peripheral arterial disease (PAD).

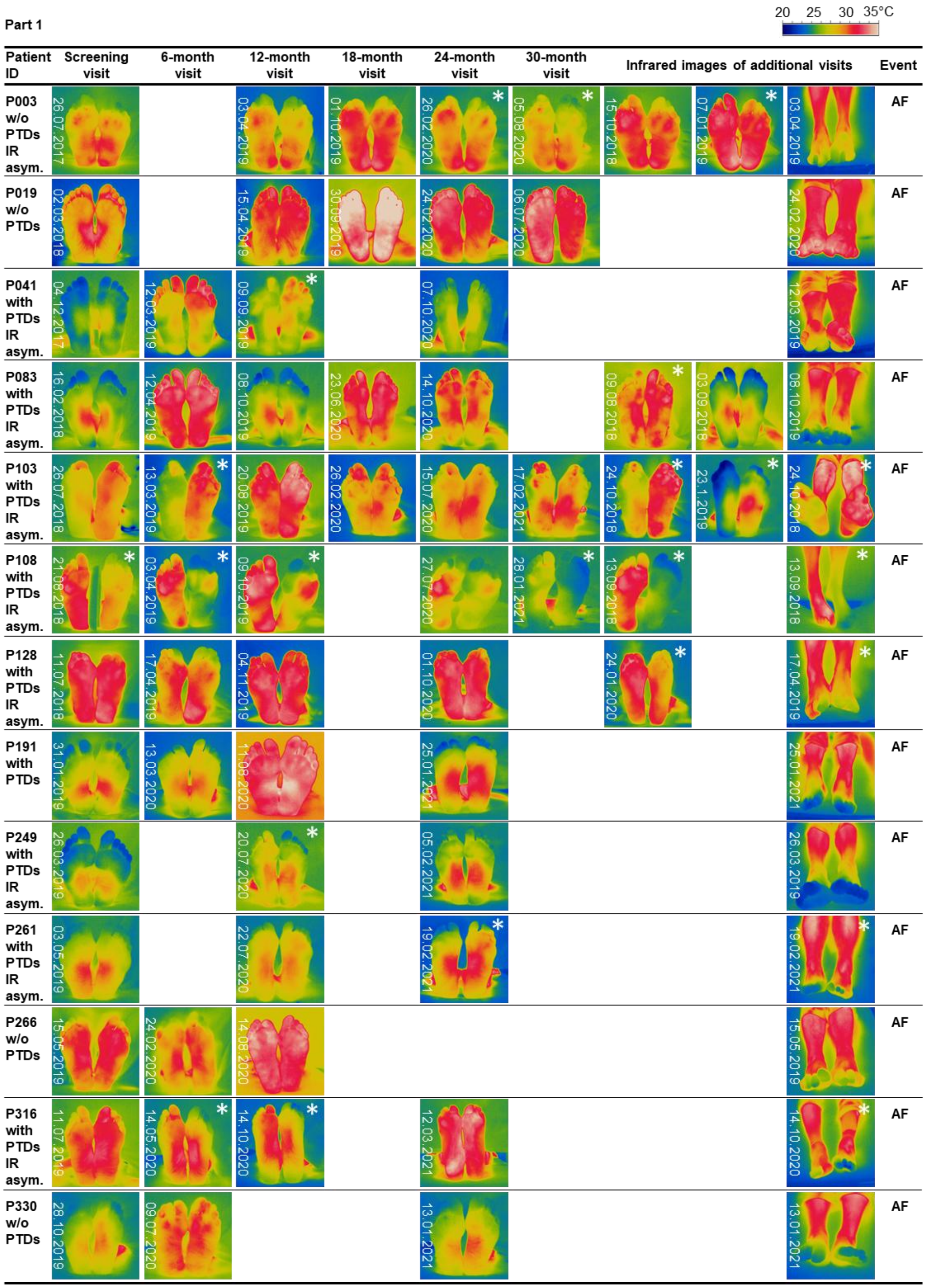

Part 2

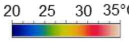

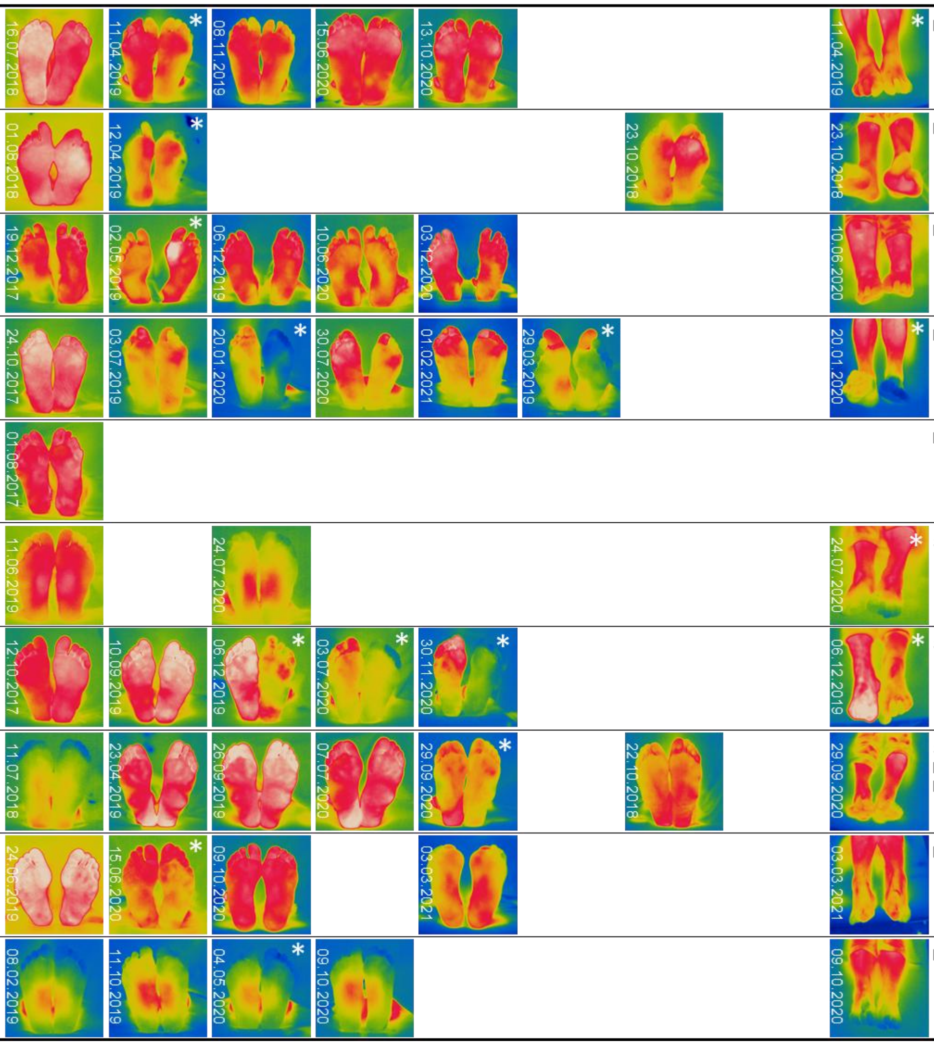

Patient ID	Screening visit	6-month visit	12-month visit	18-month visit	24-month visit	30-month visit	Infrared images of additional visits		Event
P135 with PTDs IR asym.	16.07.2018	11.04.2019 *	08.11.2019	15.06.2020	13.10.2020			11.04.2019 *	PAD
P154 with PTDs IR asym.	01.08.2018	12.04.2019 *					23.10.2018	23.10.2018	PAD
P176 with PTDs IR asym.	19.12.2017	02.05.2019 *	06.12.2019	10.06.2020	03.12.2020			10.06.2020	PAD
P183 with PTDs IR asym.	24.10.2017	03.07.2019	20.01.2020 *	30.07.2020	01.02.2021	29.03.2019 *		20.01.2020 *	PAD
P032 w/o PTDs	01.08.2017								PAD
P282 with PTDs IR asym.	11.06.2019		24.07.2020					24.07.2020 *	Stroke
P174 with PTDs IR asym.	12.10.2017	10.09.2019	06.12.2019 *	03.07.2020 *	30.11.2020 *			06.12.2019 *	Stroke
P126 with PTDs IR asym.	11.07.2018	23.04.2019	26.09.2019	07.07.2020	29.09.2020 *		22.10.2018	29.09.2020	Stroke PE PAD
P298 w/o PTDs IR asym.	24.06.2019	15.06.2020 *	09.10.2020		03.03.2021			03.03.2021	PE
	08.02.2019	11.10.2019	04.05.2020 *	09.10.2020				09.10.2020	PE

2.3 Suppl. Figure 3. Infrared thermography in intervention participants without atrial fibrillation and thromboembolic events (n = 95).

Nineteen of 95 participants (20%) showed asymmetric infrared patterns; corresponding images are marked with a white star.

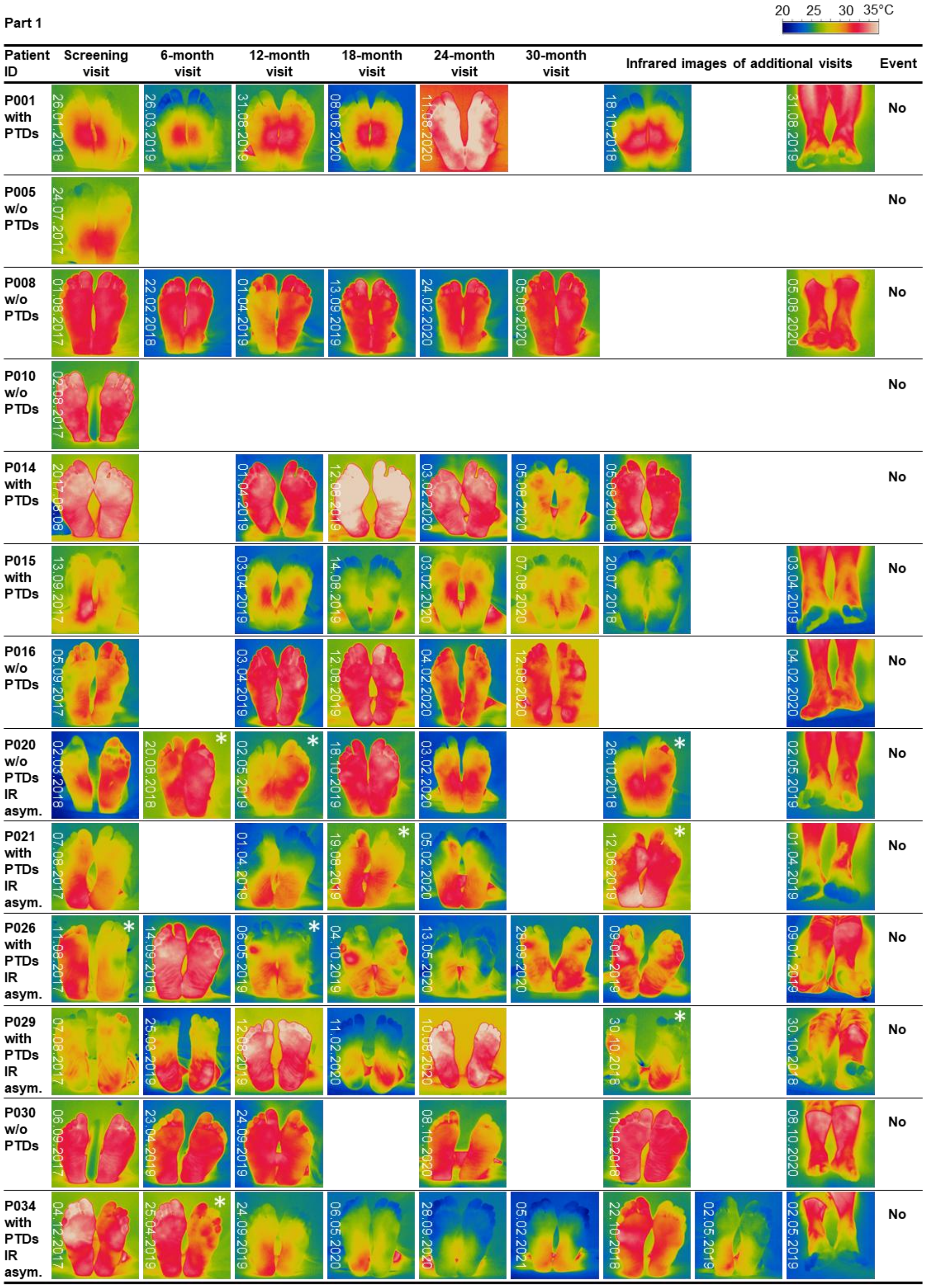

Part 2

20 25 30 35°C

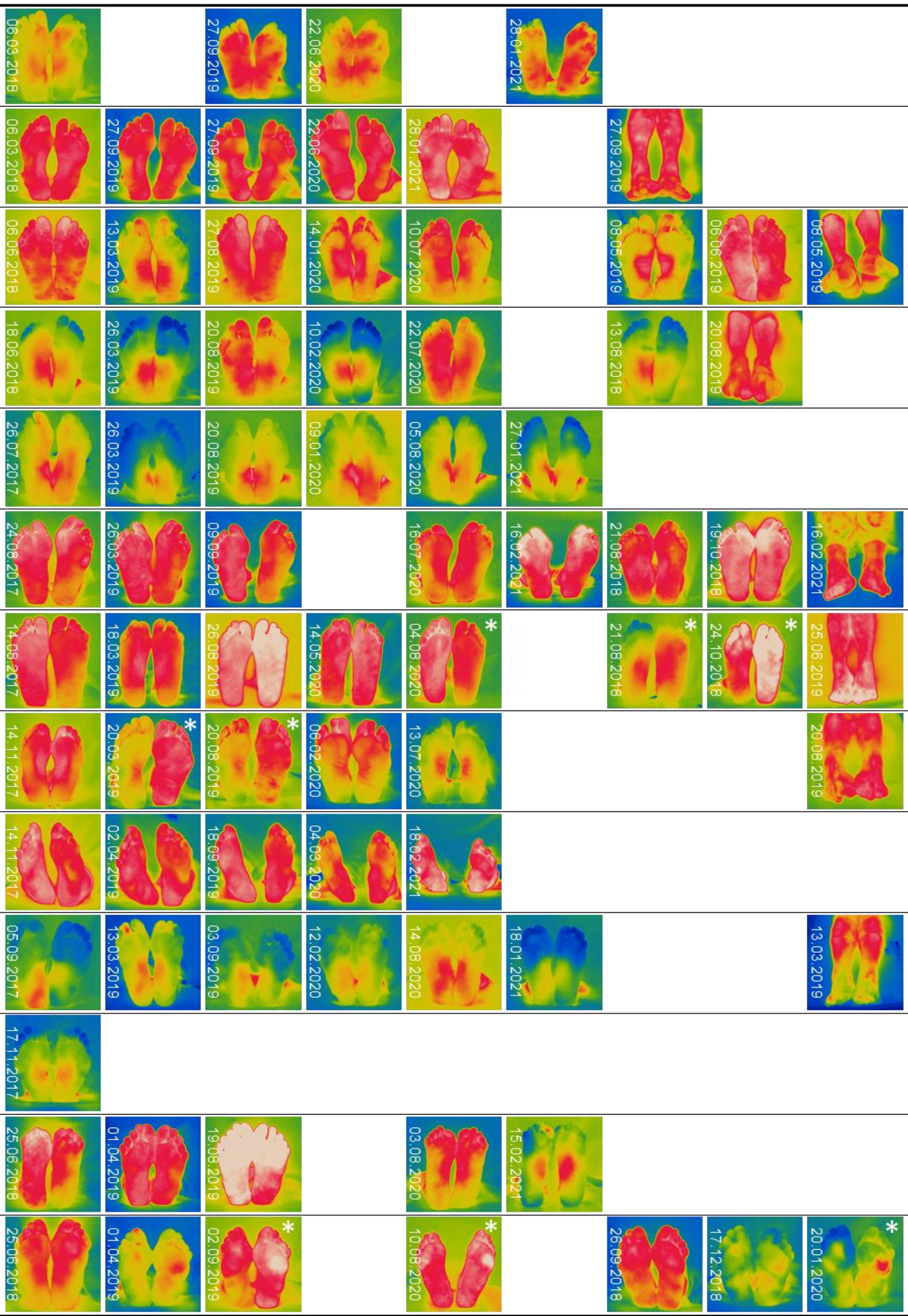

Patient ID	Screening visit	6-month visit	12-month visit	18-month visit	24-month visit	30-month visit	Infrared images of additional visits			Event
P047 w/o PTDs	06.03.2018		27.09.2019	22.06.2020		28.01.2021				No
P058 with PTDs	06.03.2018	27.09.2019	27.09.2019	22.06.2020	28.01.2021		27.09.2019			No
P061 w/o PTDs	06.06.2018	13.03.2019	27.08.2019	14.01.2020	10.07.2020		08.05.2019	06.06.2019	08.05.2019	No
P062 with PTDs	18.06.2018	26.03.2019	20.08.2019	10.02.2020	22.07.2020		13.08.2018	20.08.2019		No
P064 w/o PTDs	26.07.2017	26.03.2019	20.08.2019	09.01.2020	05.08.2020	27.01.2021				No
P065 with PTDs	24.08.2017	26.03.2019	09.09.2019		16.07.2020	16.02.2021	21.08.2018	19.10.2018	16.02.2021	No
P068 w/o PTDs IR asym.	14.08.2017	18.03.2019	26.08.2019	14.05.2020	04.08.2020 *		21.08.2018 *	24.10.2018 *	25.06.2019	No
P070 with PTDs IR asym.	14.11.2017	20.03.2019 *	20.08.2019 *	06.02.2020	13.07.2020				20.08.2019	No
P073 w/o PTDs	14.11.2017	02.04.2019	18.09.2019	04.03.2020	18.02.2021					No
P075 with PTDs	05.09.2017	13.03.2019	03.09.2019	12.02.2020	14.08.2020	18.01.2021			13.03.2019	No
P077 w/o PTDs	17.11.2017									No
P085 w/o PTDs	25.06.2018	01.04.2019	19.08.2019		03.08.2020	15.02.2021				No
	25.06.2018	01.04.2019	02.09.2019 *		10.08.2020 *		26.09.2018	17.12.2018	20.01.2020 *	No

Part 3

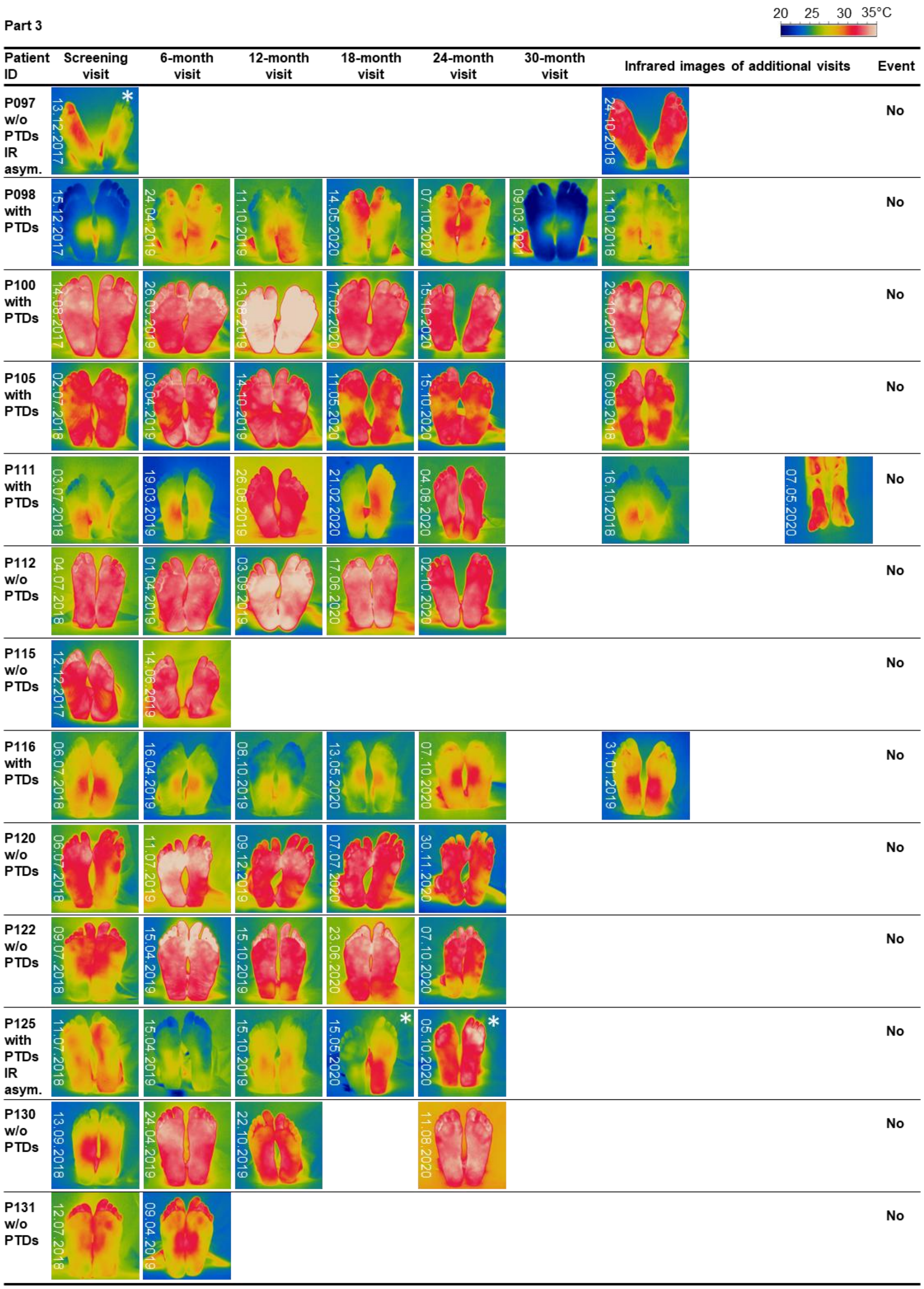

20 25 30 35°C
Patient ID
Screening visit
6-month visit
12-month visit
18-month visit
24-month visit
30-month visit
Infrared images of additional visits
Event
P097 w/o PTDs IR asym.
13.12.2017
24.10.2018
No
P098 with PTDs
15.12.2017
24.04.2019
11.10.2019
14.05.2020
07.10.2020
09.03.2021
11.10.2018
No
P100 with PTDs
14.08.2017
26.03.2019
13.08.2019
17.02.2020
15.10.2020
23.10.2018
No
P105 with PTDs
02.07.2018
03.04.2019
14.10.2019
11.05.2020
15.10.2020
06.09.2018
No
P111 with PTDs
03.07.2018
19.03.2019
26.08.2019
21.02.2020
04.08.2020
16.10.2018
07.05.2020
No
P112 w/o PTDs
04.07.2018
01.04.2019
03.09.2019
17.06.2020
02.10.2020
No
P115 w/o PTDs
12.12.2017
14.06.2019
No
P116 with PTDs
06.07.2018
16.04.2019
08.10.2019
13.05.2020
07.10.2020
31.01.2019
No
P120 w/o PTDs
06.07.2018
11.07.2019
09.12.2019
07.07.2020
30.11.2020
No
P122 w/o PTDs
09.07.2018
15.04.2019
15.10.2019
23.06.2020
07.10.2020
No
P125 with PTDs IR asym.
11.07.2018
15.04.2019
15.10.2019
15.05.2020
05.10.2020
No
P130 w/o PTDs
13.09.2018
24.04.2019
22.10.2019
11.08.2020
No
P131 w/o PTDs
12.07.2018
09.04.2019
No

Part 4

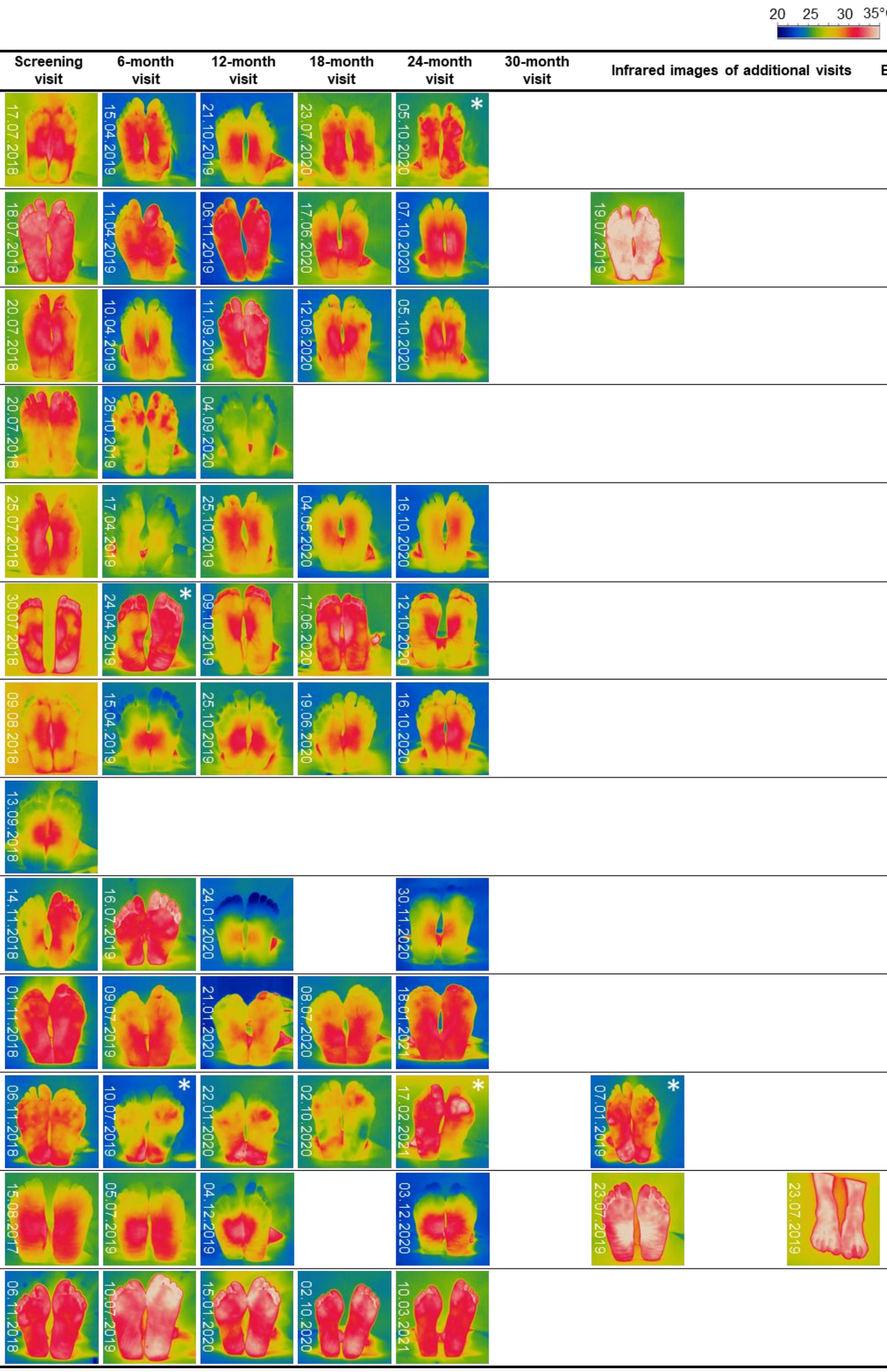

Patient ID	Screening visit	6-month visit	12-month visit	18-month visit	24-month visit	30-month visit	Infrared images of additional visits	Event
P138 with PTDs IR asym.	17.07.2018	15.04.2019	21.10.2019	23.07.2020	05.10.2020 *			No
P140 with PTDs	18.07.2018	11.04.2019	06.11.2019	17.06.2020	07.10.2020		19.07.2019	No
P143 w/o PTDs	20.07.2018	10.04.2019	11.09.2019	12.06.2020	05.10.2020			No
P144 with PTDs	20.07.2018	28.10.2019	04.09.2020					No
P146 with PTDs	25.07.2018	17.04.2019	25.10.2019	04.05.2020	16.10.2020			No
P150 with PTDs IR asym.	30.07.2018	24.04.2019 *	09.10.2019	17.06.2020	12.10.2020			No
P159 w/o PTDs	09.08.2018	15.04.2019	25.10.2019	19.06.2020	16.10.2020			No
P163 w/o PTDs	13.09.2018							No
P165 with PTDs	14.11.2018	16.07.2019	24.01.2020		30.11.2020			No
P170 w/o PTDs	01.11.2018	09.07.2019	21.01.2020	08.07.2020	18.01.2021			No
P173 with PTDs IR asym.	06.11.2018	10.07.2019 *	22.01.2020	02.10.2020	17.02.2021 *		07.01.2019 *	No
P175 with PTDs	15.08.2017	05.07.2019	04.12.2019		03.12.2020		23.07.2019; 23.07.2019	No
P178 with PTDs	06.11.2018	10.07.2019	15.01.2020	02.10.2020	10.03.2021			No

Part 5

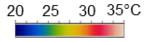

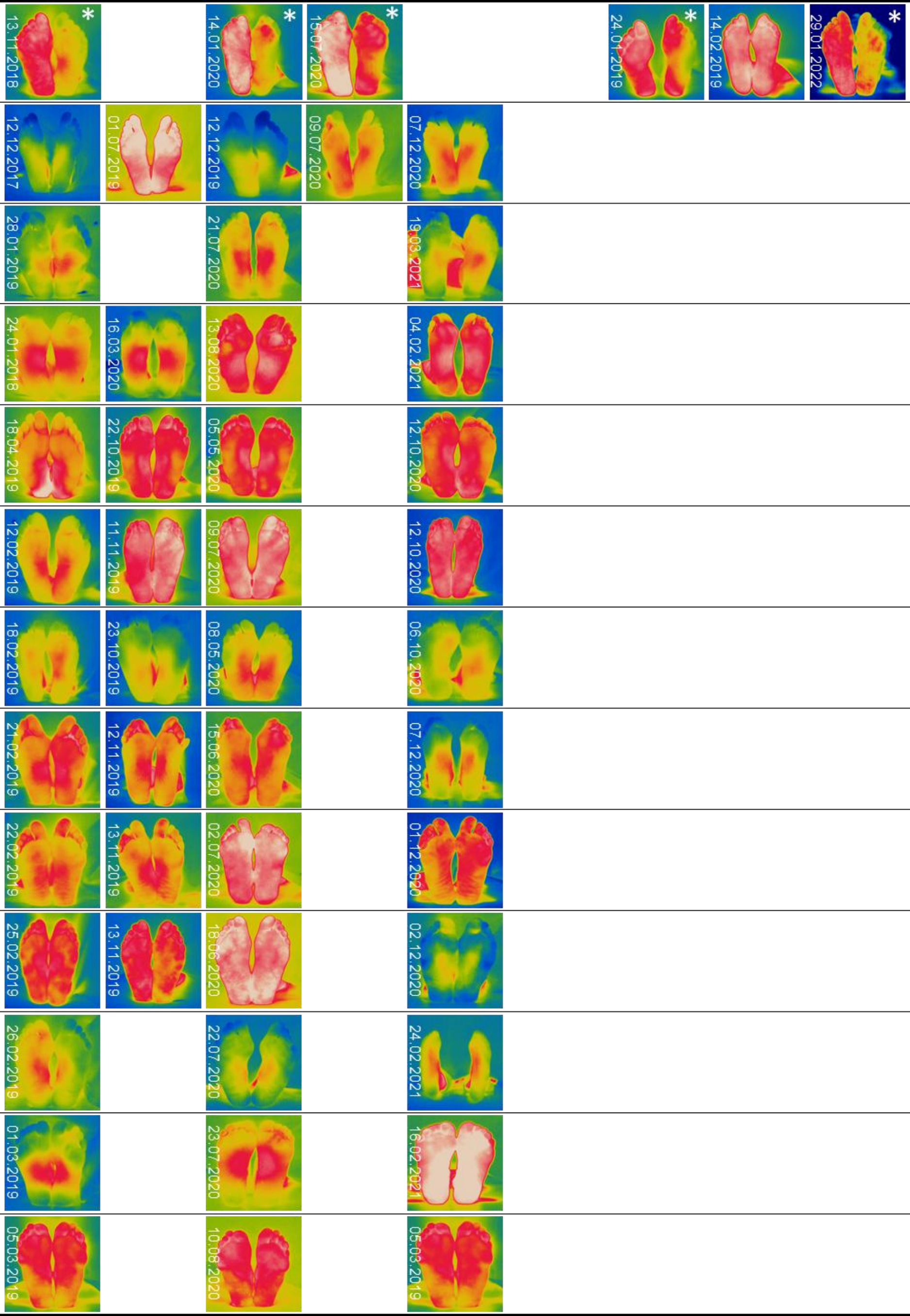

Patient ID	Screening visit	6-month visit	12-month visit	18-month visit	24-month visit	30-month visit	Infrared images of additional visits			Event
P181 with PTDs IR asym.	13.11.2018 *		14.01.2020 *	15.07.2020 *			24.01.2019 *	14.02.2019	29.01.2022 *	No
P184 with PTDs	12.12.2017	01.07.2019	12.12.2019	09.07.2020	07.12.2020					No
P187 w/o PTDs	28.01.2019		21.07.2020		19.03.2021					No
P193 w/o PTDs	24.01.2018	16.03.2020	13.08.2020		04.02.2021					No
P207 w/o PTDs	18.04.2019	22.10.2019	05.05.2020		12.10.2020					No
P208 with PTDs	12.02.2019	11.11.2019	09.07.2020		12.10.2020					No
P216 with PTDs	18.02.2019	23.10.2019	08.05.2020		06.10.2020					No
P221 w/o PTDs	21.02.2019	12.11.2019	15.06.2020		07.12.2020					No
P222 with PTDs	22.02.2019	13.11.2019	02.07.2020		01.12.2020					No
P223 with PTDs	25.02.2019	13.11.2019	18.06.2020		02.12.2020					No
P226 w/o PTDs	26.02.2019		22.07.2020		24.02.2021					No
P229 with PTDs	01.03.2019		23.07.2020		16.02.2021					No
P230 w/o PTDs	05.03.2019		10.08.2020		05.03.2019					No

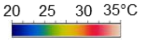

Part 6

Patient ID	Screening visit	6-month visit	12-month visit	18-month visit	24-month visit	30-month visit	Infrared images of additional visits	Event
P232 with PTDs	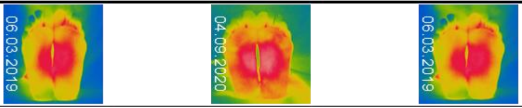							No
P234 w/o PTDs	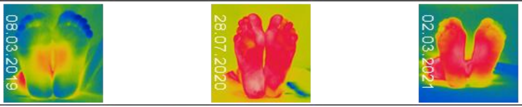							No
P239 with PTDs	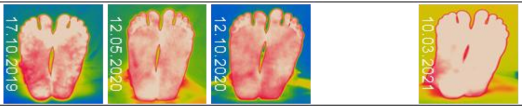							No
P242 w/o PTDs	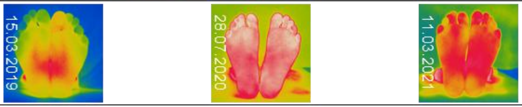							No
P250 w/o PTDs	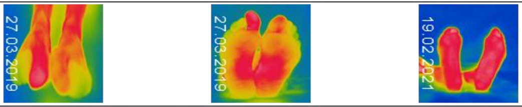							No
P254 with PTDs	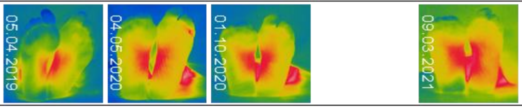							No
P255 with PTDs	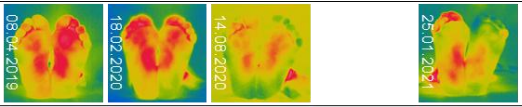							No
P276 with PTDs	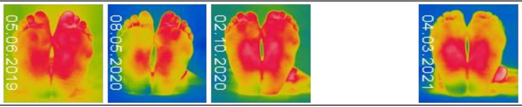							No
P277 with PTDs	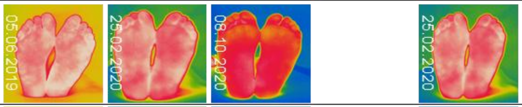							No
P283 with PTDs IR asym.	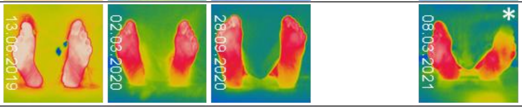							No
P285 w/o PTDs	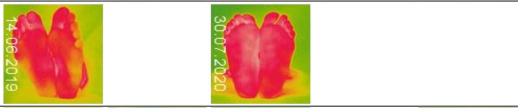							No
P290 w/o PTDs	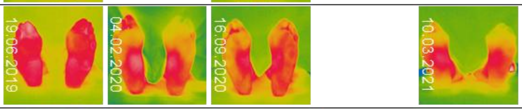							No
P291 w/o PTDs	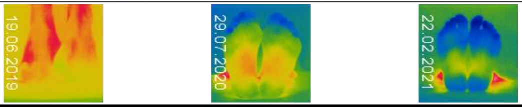							No

Part 7

20 25 30 35°C

Patient ID	Screening visit	6-month visit	12-month visit	18-month visit	24-month visit	30-month visit	Infrared images of additional visits	Event
**P295 w/o PTDs**	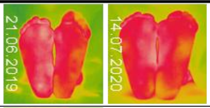				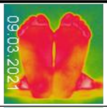			**No**
**P296 w/o PTDs**	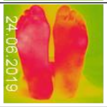		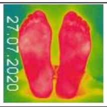		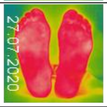			**No**
**P299 with PTDs**	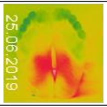	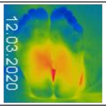	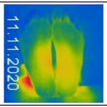		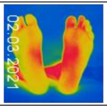			**No**
**P301 w/o PTDs**	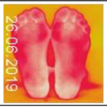		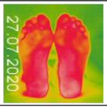		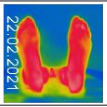			**No**
**P304 w/o PTDs**	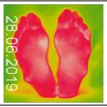		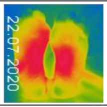					**No**
**P308 w/o PTDs**	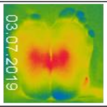		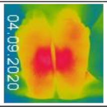		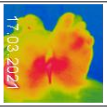			**No**
**P310 with PTDs IR asym.**	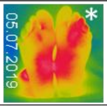		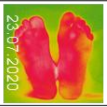		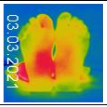			**No**
**P311 w/o PTDs**	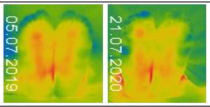							**No**
**P320 with PTDs IR asym.**	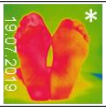	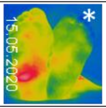	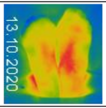		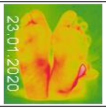		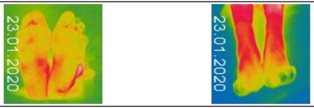	**No**
**P323 with PTDs IR asym.**	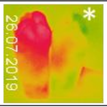		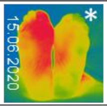		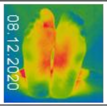			**No**
**P326 w/o PTDs**	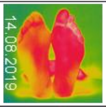		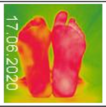		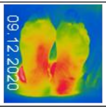		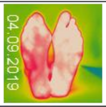 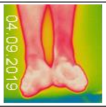	**No**
**P333 with PTDs IR asym.**	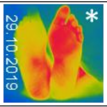		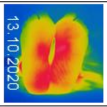		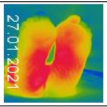			**No**
**P337 w/o PTDs**	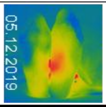	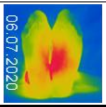			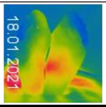			**No**

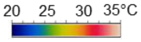

Part 8

Patient ID	Screening visit	6-month visit	12-month visit	18-month visit	24-month visit	30-month visit	Infrared images of additional visits	Event
**P338 w/o PTDs**	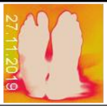		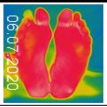		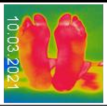			**No**
**P341 with PTDs**	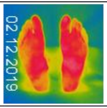	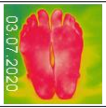			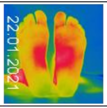			**No**
**P348 with PTDs**	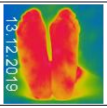	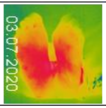			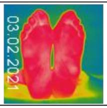			**No**
**P350 with PTDs**	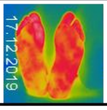	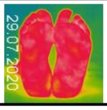			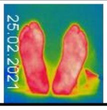			**No**

2.4 Suppl. Figure 4. Infrared thermography in control participants with atrial fibrillation and thromboembolic events (n = 22).

Fifteen of 22 participants (68%) exhibited asymmetric infrared patterns; corresponding images are marked with a white star. Events include atrial fibrillation (AF), stroke, pulmonary embolism (PE), and peripheral arterial disease (PAD).

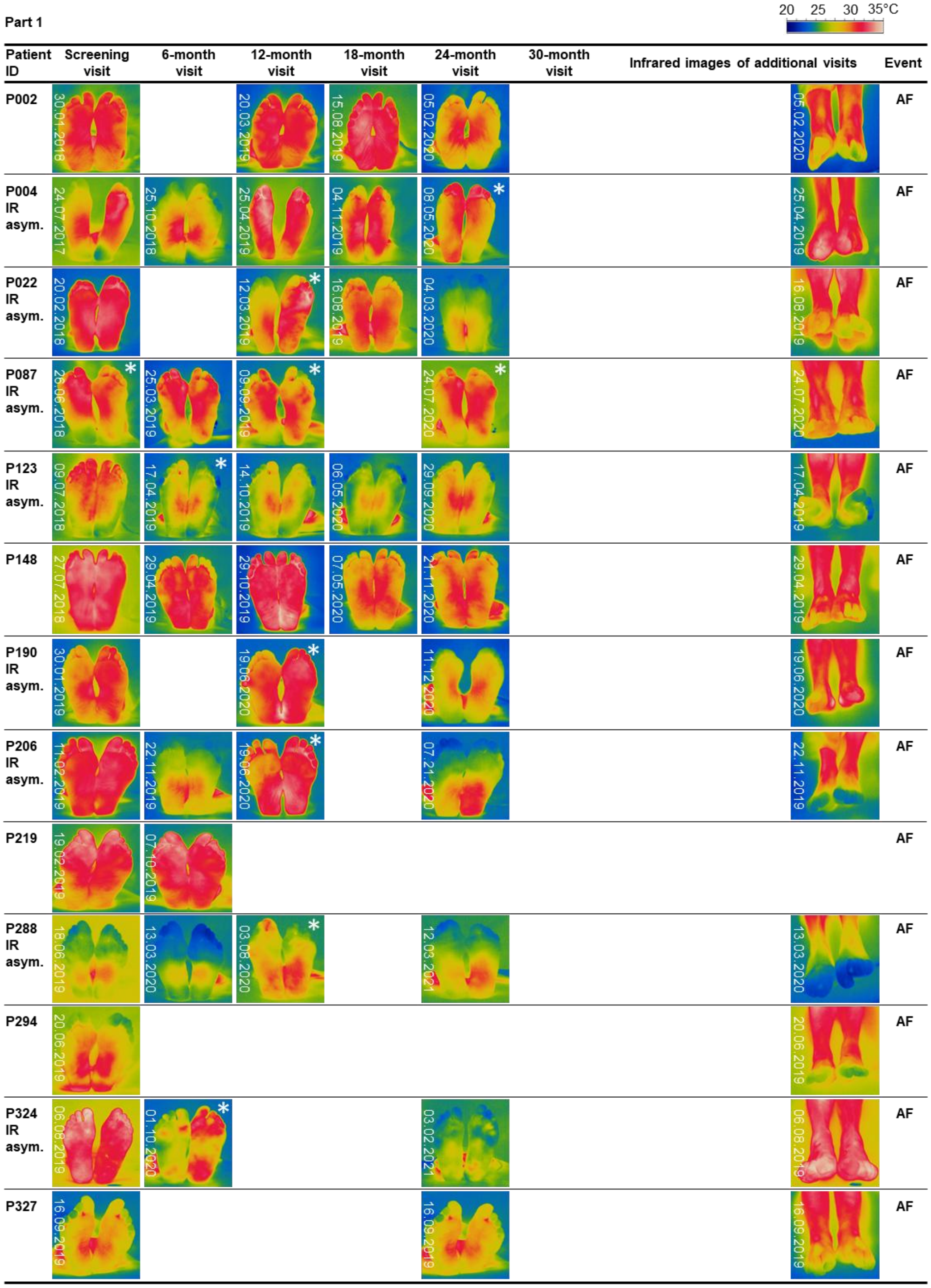

Part 2

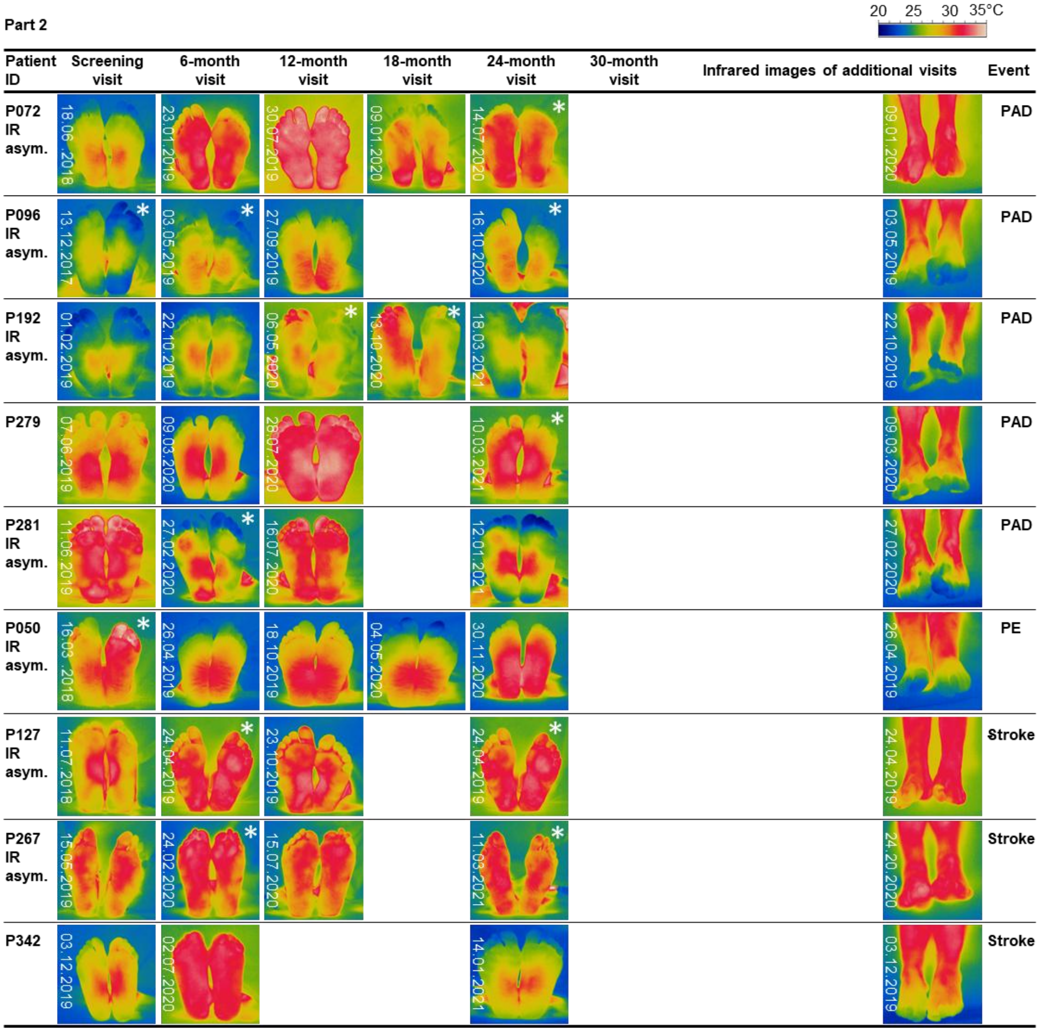
20 25 30 35°C
Patient ID
Screening visit
6-month visit
12-month visit
18-month visit
24-month visit
30-month visit
Infrared images of additional visits
Event
P072 IR asym.
PAD
P096 IR asym.
PAD
P192 IR asym.
PAD
P279
PAD
P281 IR asym.
PAD
P050 IR asym.
PE
P127 IR asym.
Stroke
P267 IR asym.
Stroke
P342
Stroke

2.5 Suppl. Figure 5. Infrared thermography in control participants without atrial fibrillation and thromboembolic events (n = 99).

Sixteen of 99 participants (16%) showed asymmetric infrared patterns; corresponding images are marked with a white star.

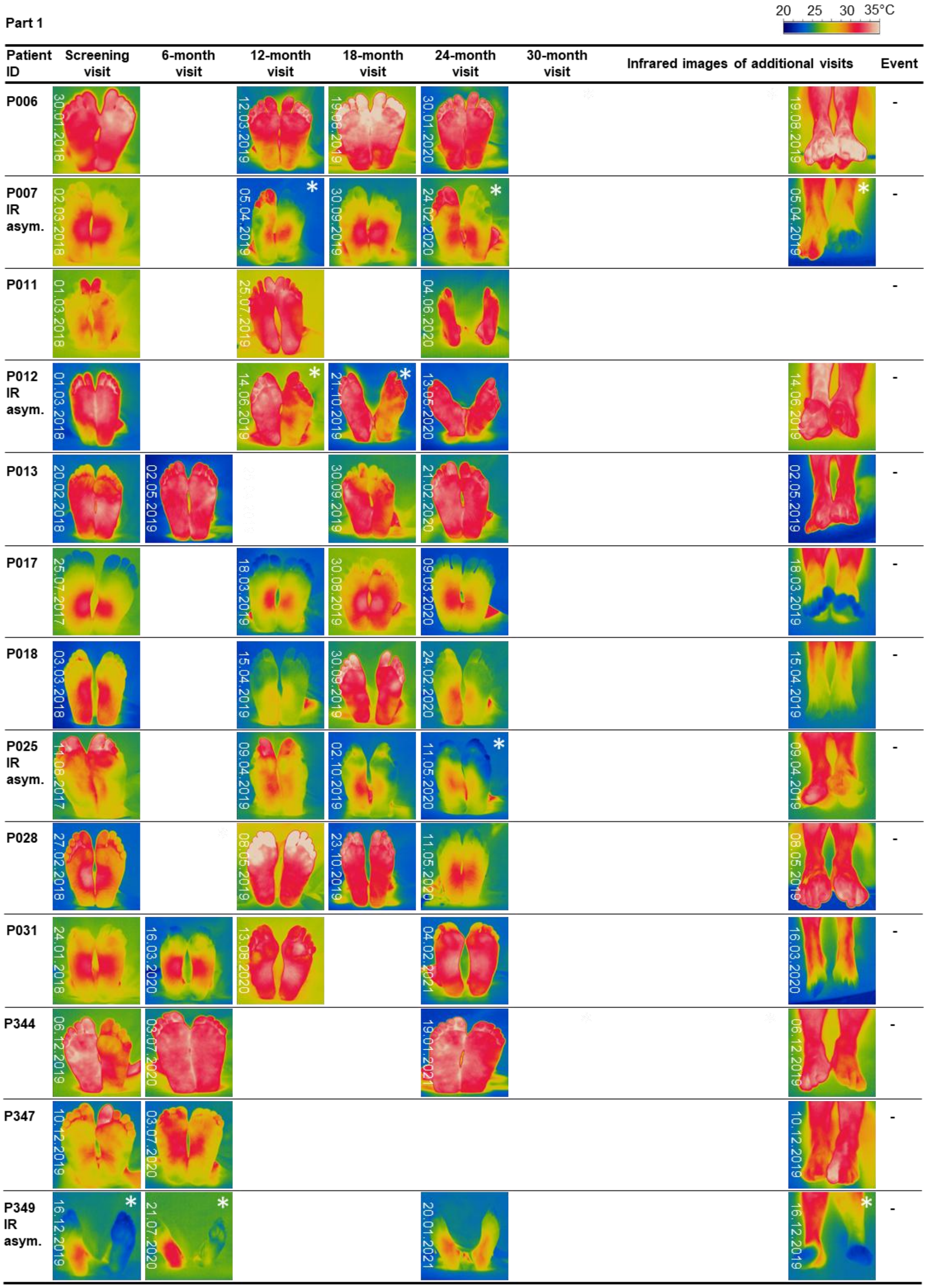

Part 2

Patient ID	Screening visit	6-month visit	12-month visit	18-month visit	24-month visit	30-month visit	Infrared images of additional visits	Event
P033	16.03.2018							-
P036 IR asym.	23.03.2018 *	16.01.2019 *	22.07.2019 *	09.01.2020 *	20.07.2020 *		22.07.2019 *	-
P042	13.02.2018	15.01.2019	06.09.2019		31.07.2020	01.10.2019	06.09.2019	-
P044	01.03 .2018	21.01.2019	24.07.2019	22.01.2020	08.07.2020		22.01.2020	-
P045	02.03 .2018	27.03.2019	28.08.2019	04.08.2020	04.08.2020		28.08.2019	-
P051 IR asym.	12.04 .2018	17.01.2019 *	26.07.2019	08.01.2020	10.07.2020		26.07.2019	-
P052	12.04 .2018	17.01.2019	26.07.2019	08.01.2020	10.07.2020		10.07.2020	-
P053	10.04 .2018		29.07.2019	22.01.2020	10.07.2020		22.01.2020	-
P060	03.05.2018	22.02.2019	29.07.2019	07.01.2020	13.07.2020	20.06.2019	20.06.2019	-
P063	18.06 .2018	23.01.2019	30.07.2019	09.01.2020	14.07.2020		09.01.2020	-
P071	20.06 .2018							-
P351	18.12.2019	04.09.2020			04.02.2021		18.12.2019	-
P307	03.07.2019		05.08.2020		13.01.2021		05.08.2020	-

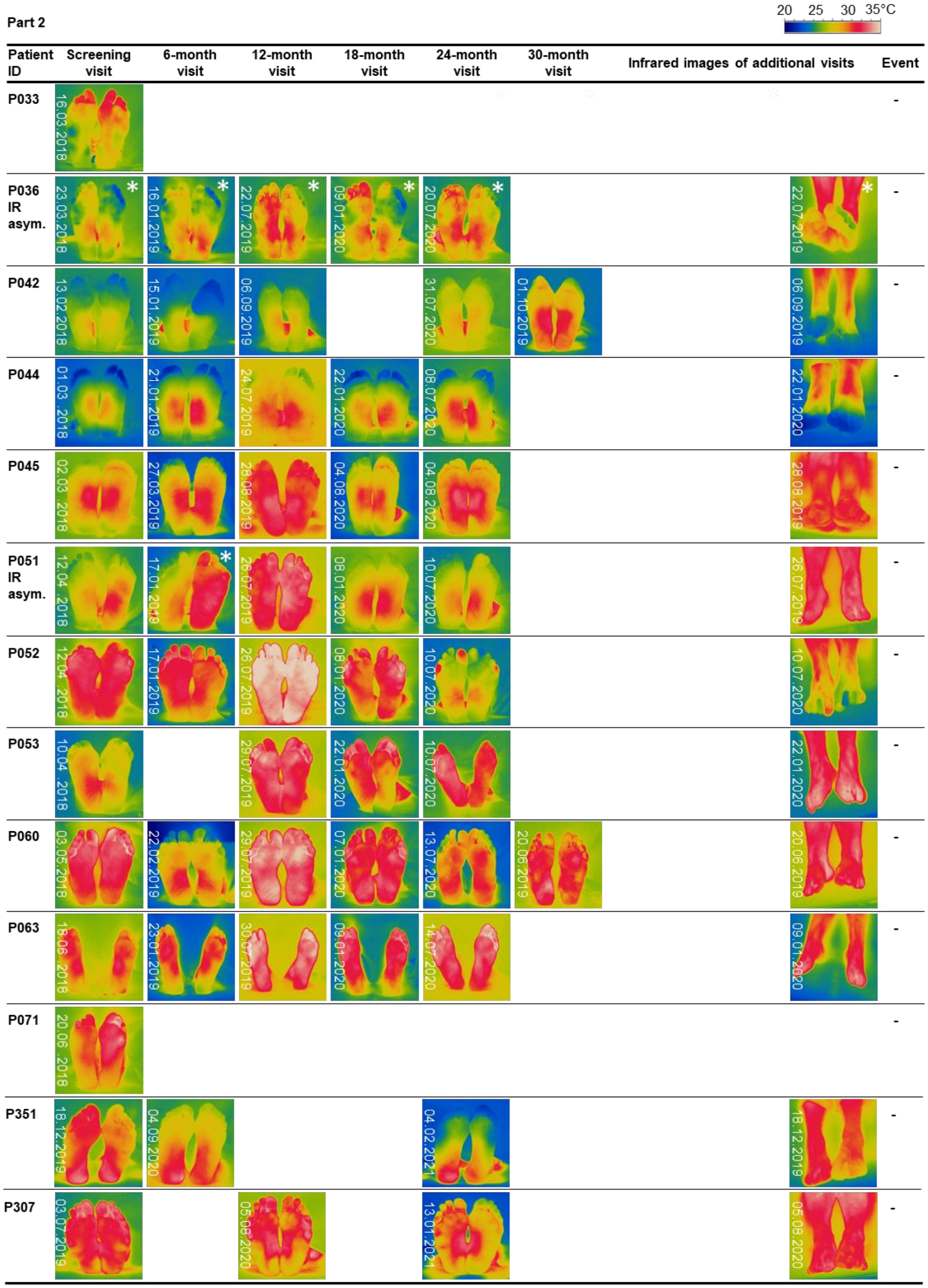

Part 3

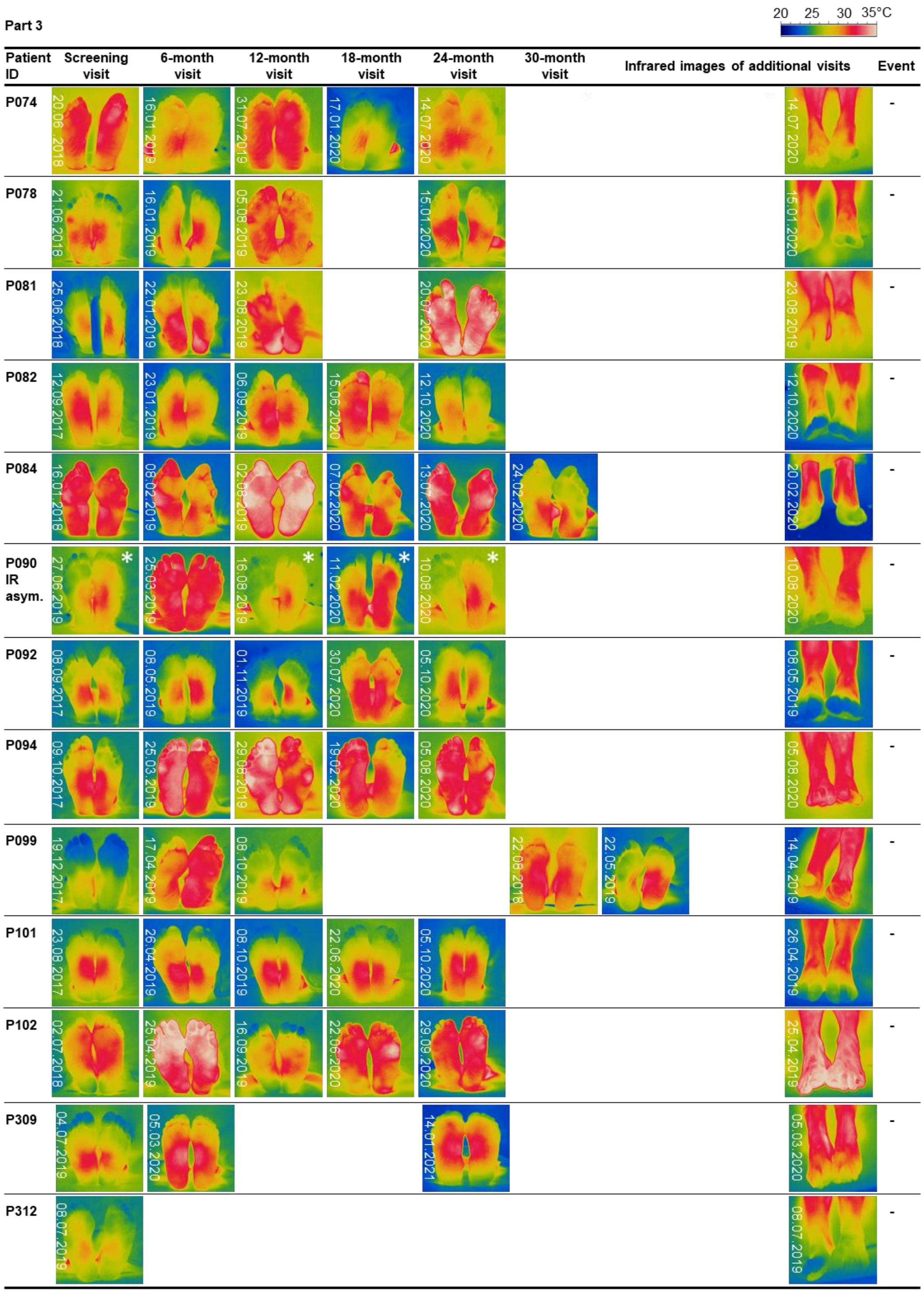

20 25 30 35°C
Patient ID
Screening visit
6-month visit
12-month visit
18-month visit
24-month visit
30-month visit
Infrared images of additional visits
Event
P074
20.06.2018
16.01.2019
31.07.2019
17.01.2020
14.07.2020
14.07.2020
-
P078
21.06.2018
16.01.2019
05.08.2019
15.01.2020
15.01.2020
-
P081
25.06.2018
22.01.2019
23.08.2019
20.07.2020
23.08.2019
-
P082
12.09.2017
23.01.2019
06.09.2019
15.06.2020
12.10.2020
12.10.2020
-
P084
16.01.2018
08.02.2019
07.02.2020
13.07.2020
24.02.2020
20.02.2020
-
P090
IR
asym.
27.06.2019
25.03.2019
16.08.2019
11.02.2020
10.08.2020
10.08.2020
-
P092
08.09.2017
08.05.2019
01.11.2019
30.07.2020
05.10.2020
08.05.2019
-
P094
09.10.2017
25.03.2019
29.08.2019
19.02.2020
05.08.2020
05.08.2020
-
P099
19.12.2017
17.04.2019
08.10.2019
22.08.2018
22.05.2019
14.04.2019
-
P101
23.08.2017
26.04.2019
08.10.2019
22.06.2020
05.10.2020
26.04.2019
-
P102
02.07.2018
25.04.2019
16.09.2019
22.06.2020
29.09.2020
25.04.2019
-
P309
04.07.2019
05.03.2020
14.01.2021
05.03.2020
-
P312
08.07.2019
08.07.2019
-

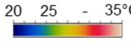

Part 4

Patient ID	Screening visit	6-month visit	12-month visit	18-month visit	24-month visit	30-month visit	Infrared images of additional visits	Event
P104	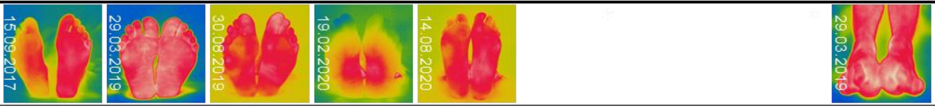							-
P106 IR asym.	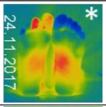							-
P107	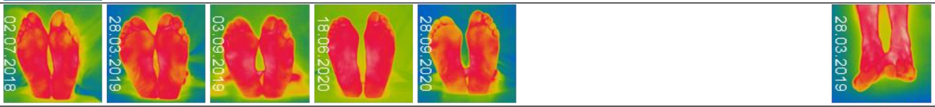							-
P109	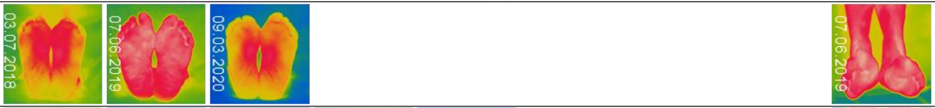							-
P114	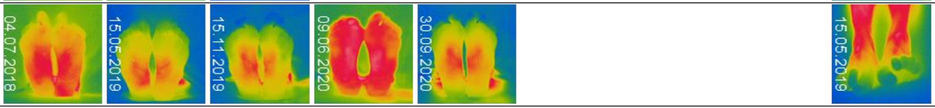							-
P117	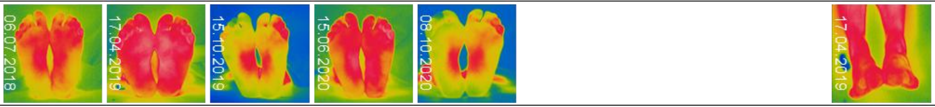							-
P121	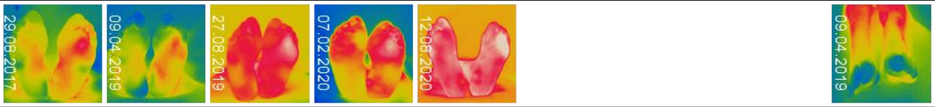							-
P124	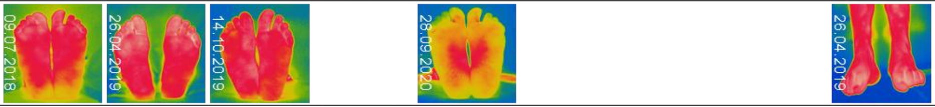							-
P129	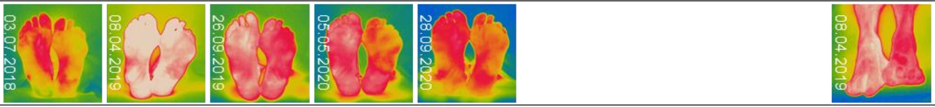							-
P132	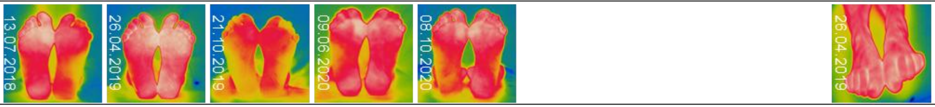							-
P133	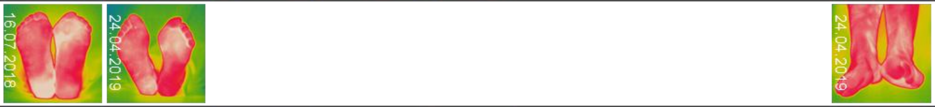							-
P314	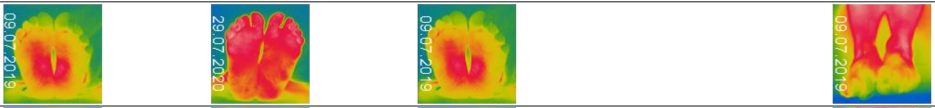							-
P319	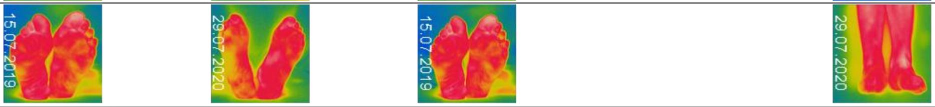							-

Part 5

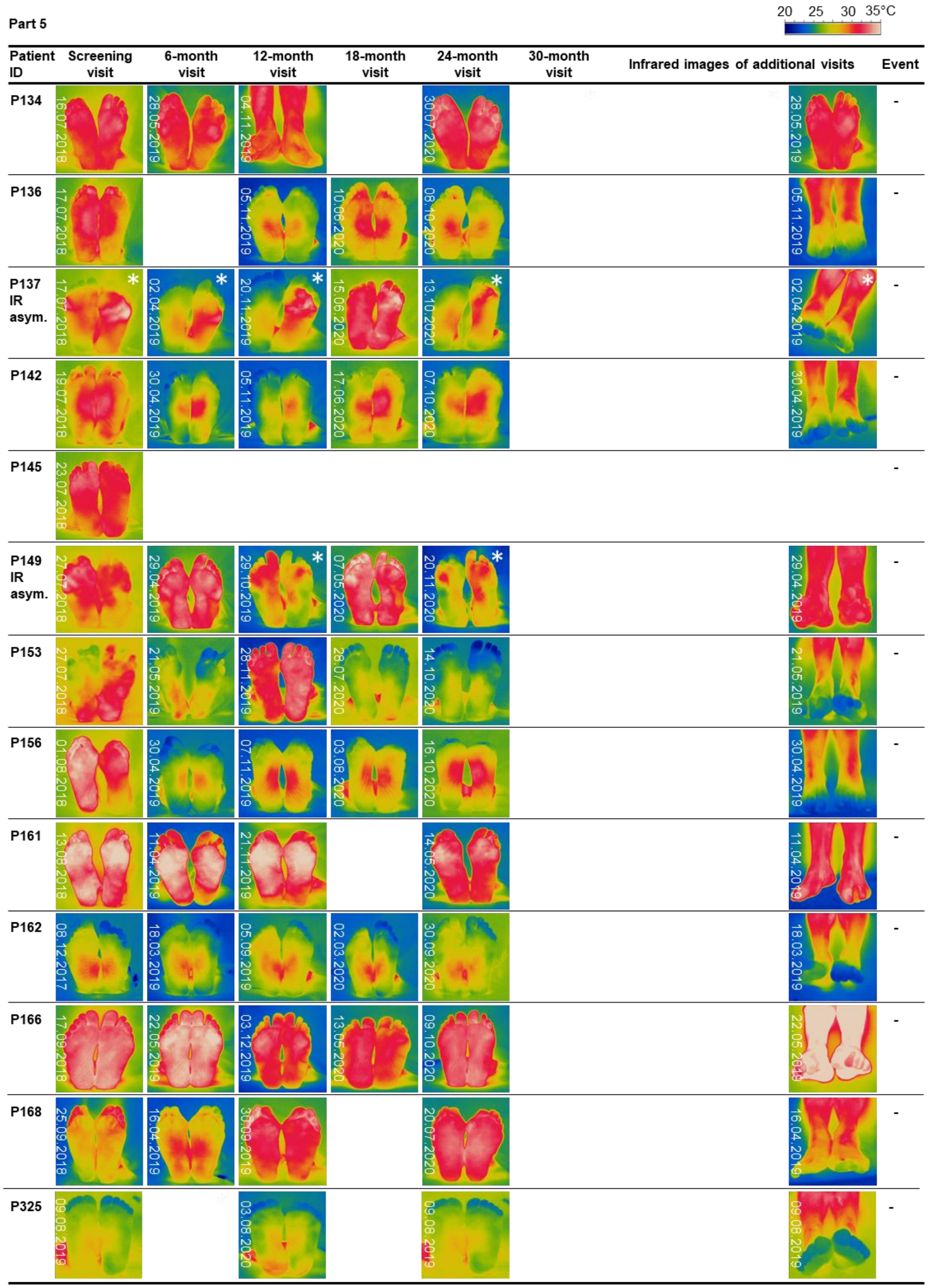
20 25 30 35°C
Patient ID
Screening visit
6-month visit
12-month visit
18-month visit
24-month visit
30-month visit
Infrared images of additional visits
Event
P134
16.07.2018
28.05.2019
04.11.2019
30.07.2020
28.05.2019
-
P136
17.07.2018
05.11.2019
10.06.2020
08.10.2020
05.11.2019
-
P137 IR asym.
17.07.2018
02.04.2019
20.11.2019
15.06.2020
13.10.2020
02.04.2019
-
P142
19.07.2018
30.04.2019
05.11.2019
17.06.2020
07.10.2020
30.04.2019
-
P145
23.07.2018
-
P149 IR asym.
29.04.2019
29.10.2019
07.05.2020
20.11.2020
29.04.2019
-
P153
27.07.2018
21.05.2019
28.11.2019
28.07.2020
14.10.2020
21.05.2019
-
P156
01.08.2018
30.04.2019
07.11.2019
03.08.2020
16.10.2020
30.04.2019
-
P161
13.08.2018
11.04.2019
21.11.2019
14.05.2020
11.04.2019
-
P162
08.12.2017
18.03.2019
05.09.2019
02.03.2020
30.09.2020
18.03.2019
-
P166
17.09.2018
22.05.2019
03.12.2019
13.05.2020
09.10.2020
22.05.2019
-
P168
25.09.2018
16.04.2019
20.07.2020
16.04.2019
-
P325
09.08.2019
03.08.2020
09.08.2020
09.08.2019
-

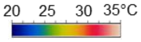

Part 6

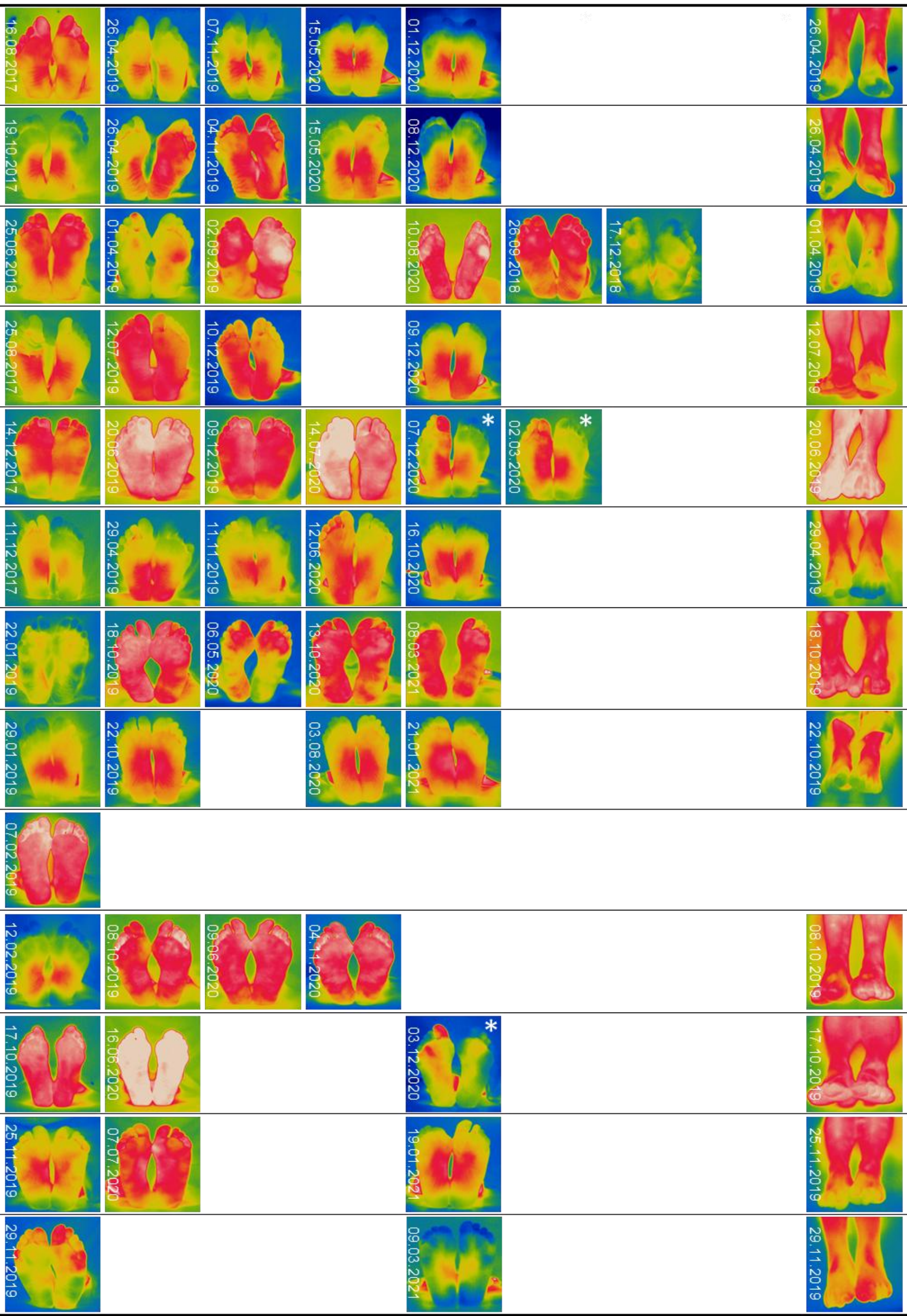

Patient ID	Screening visit	6-month visit	12-month visit	18-month visit	24-month visit	30-month visit	Infrared images of additional visits		Event
P169	16.08.2017	26.04.2019	07.11.2019	15.05.2020	01.12.2020			26.04.2019	-
P171	19.10.2017	26.04.2019	04.11.2019	15.05.2020	08.12.2020			26.04.2019	-
P172	25.06.2018	01.04.2019	02.09.2019		10.08.2020	26.09.2018	17.12.2018	01.04.2019	-
P179	25.08.2017	12.07.2019	10.12.2019		09.12.2020			12.07.2019	-
P180 IR asym.	14.12.2017	20.06.2019	09.12.2019	14.07.2020	07.12.2020 *	02.03.2020 *		20.06.2019	-
P182	11.12.2017	29.04.2019	11.11.2019	12.06.2020	16.10.2020			29.04.2019	-
P185	22.01.2019	18.10.2019	06.05.2020	13.10.2020	08.03.2021			18.10.2019	-
P188	29.01.2019	22.10.2019		03.08.2020	21.01.2021			22.10.2019	-
P200	07.02.2019								-
P209	12.02.2019	08.10.2019	09.06.2020	04.11.2020				08.10.2019	-
P331 IR asym.	17.10.2019	16.06.2020			03.12.2020 *			17.10.2019	-
P334	25.11.2019	07.07.2020			19.01.2021			25.11.2019	-
P339	29.11.2019				09.03.2021			29.11.2019	-

Part 7

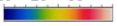

Patient ID	Screening visit	6-month visit	12-month visit	18-month visit	24-month visit	30-month visit	Infrared images of additional visits	Event
P211	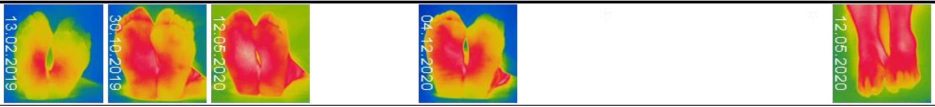 13.02.2019	30.10.2019	12.05.2020		04.12.2020		12.05.2020	-
P213 IR asym.	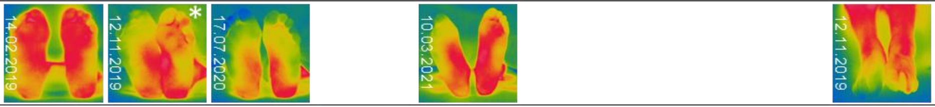 14.02.2019	12.11.2019 *	17.07.2020		10.03.2021		12.11.2019	-
P218	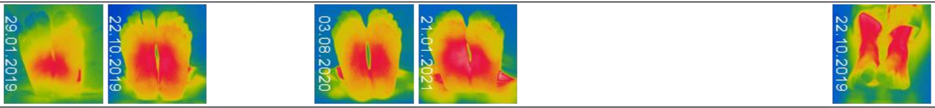 29.01.2019	22.10.2019		03.08.2020	21.01.2021		22.10.2019	-
P227	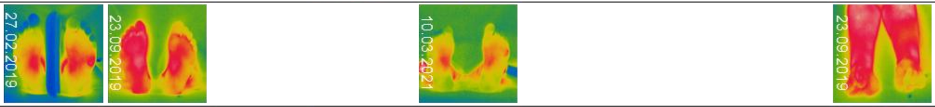 27.02.2019	23.09.2019			10.03.2021		23.09.2019	-
P228	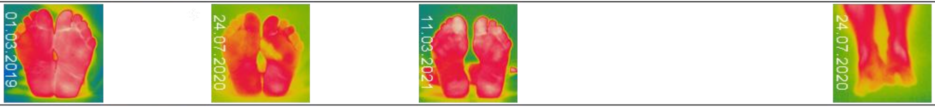 01.03.2019		24.07.2020		11.03.2021		24.07.2020	-
P233	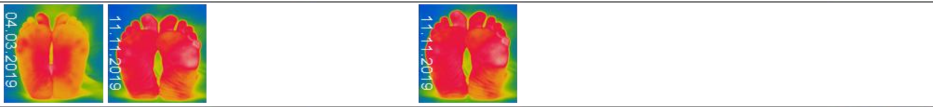 04.03.2019	11.11.2019			11.11.2019			-
P236	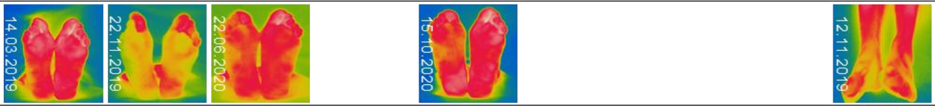 14.03.2019	22.11.2019	22.06.2020		15.10.2020		12.11.2019	-
P237 IR asym.	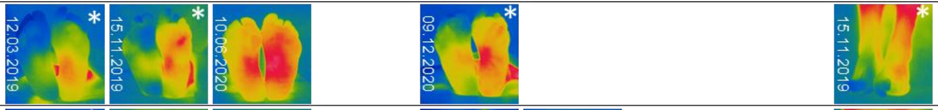 12.03.2019 *	15.11.2019 *	10.06.2020		09.12.2020 *		15.11.2019 *	-
P245 IR asym.	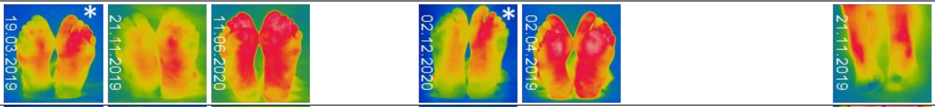 19.03.2019 *	21.11.2019	11.06.2020		02.12.2020 *	02.04.2019	21.11.2019	-
P246	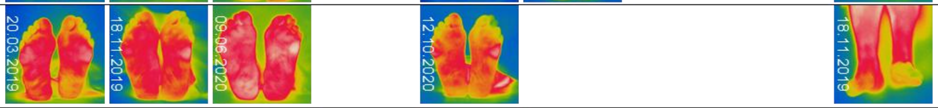 20.03.2019	18.11.2019	09.06.2020		12.10.2020		18.11.2019	-
P251	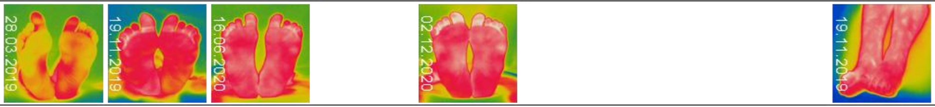 28.03.2019	19.11.2019	16.06.2020		02.12.2020		19.11.2019	-
P253	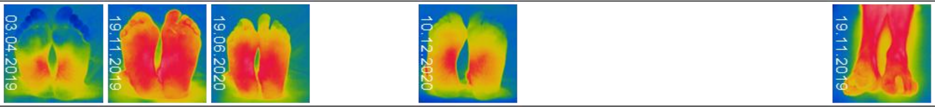 03.04.2019	19.11.2019	19.06.2020		10.12.2020		19.11.2019	-
P343	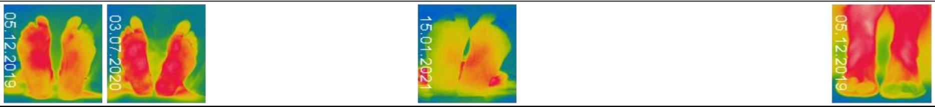 05.12.2019	03.07.2020			15.01.2021		05.12.2019	-

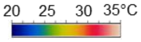

Part 8

Patient ID	Screening visit	6-month visit	12-month visit	18-month visit	24-month visit	30-month visit	Infrared images of additional visits	Event
**P259**	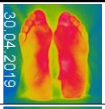		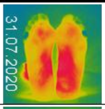		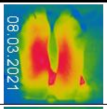		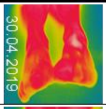	-
**P260**	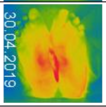		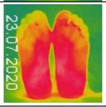		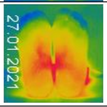		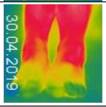	-
**P273 IR asym.**	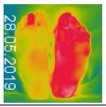	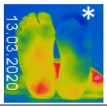	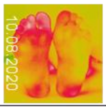		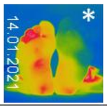		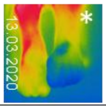	-
**P286**	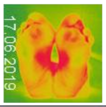	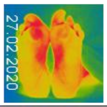	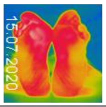		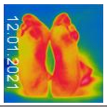		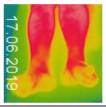	-
**P287**	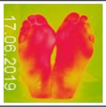	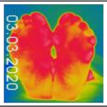	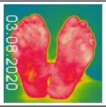		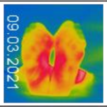		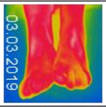	-
**P289**	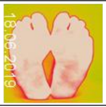		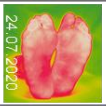		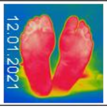		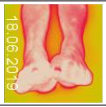	-
**P300**	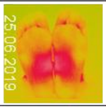	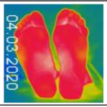	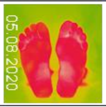		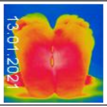		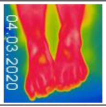	-
**P306**	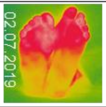		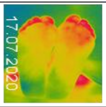		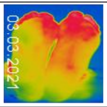		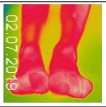	-

2.6 Suppl. Figure 6. Preprocessing of plantar temperature recordings.

(a) Visualization of plantar temperature values collected from 133 patients in the intervention groups (n=80176). (b) Stepwise data cleaning with exclusion of invalidate recordings marked with numbers on subplots.

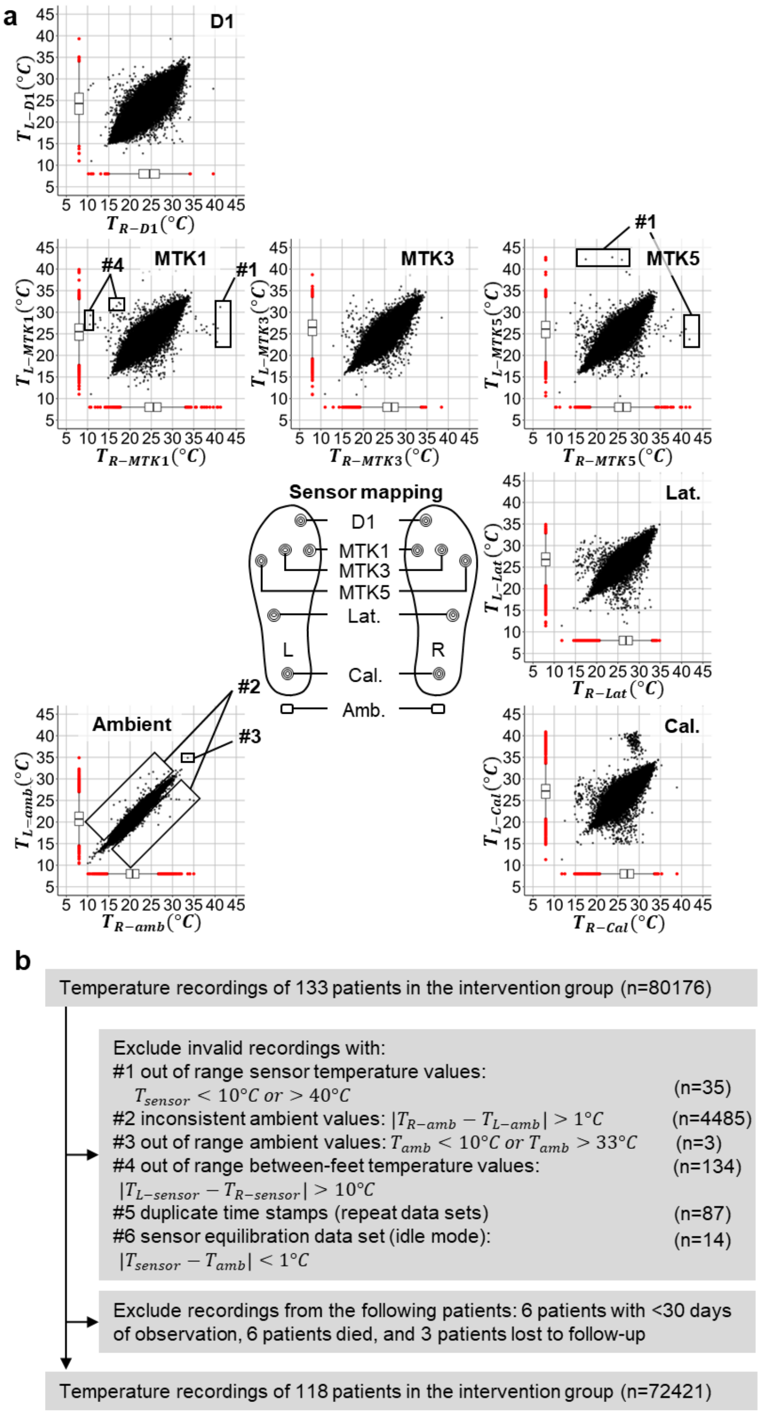

3 Smart Prevent Diabetic Feet Study protocol

The study protocol and initial findings of the original randomized controlled trial (Smart Prevent Diabetic Feet Study) have been published:

Ming A, Walter I, Alhajjar A, Leuckert M, Mertens PR. Study protocol for a randomized controlled trial to test for preventive effects of diabetic foot ulceration by telemedicine that includes sensor-equipped insoles combined with photo documentation. Trials. 2019 Aug 22;20(1):521. doi: 10.1186/s13063-019-3623-x.

Ming A, Alhajjar A, Walter I, Piehler C, Hoetzsch J, Leuckert M, Clemens V, Petrow A, Siddiquee IM, Scurt FG, Isermann B, Mertens PR. Telemedical Monitoring of Plantar Temperature in Diabetic Patients at Risk of Foot Ulcers. Dtsch Arztebl Int. 2024 Jan 12;121(1):9-16. doi: 10.3238/arztebl.m2023.0229.

4 STROBE checklist

STROBE Statement—checklist of items that should be included in reports of observational studies

	Item No	Recommendation	Page No
**Title and abstract**	1	(*a*) Indicate the study's design with a commonly used term in the title or the abstract	1
		(*b*) Provide in the abstract an informative and balanced summary of what was done and what was found	3
**Introduction**			
Background/rationale	2	Explain the scientific background and rationale for the investigation being reported	5, 6
Objectives	3	State specific objectives, including any prespecified hypotheses	7
**Methods**			
Study design	4	Present key elements of study design early in the paper	19
Setting	5	Describe the setting, locations, and relevant dates, including periods of recruitment, exposure, follow-up, and data collection	19, 20
Participants	6	(*a*) *Cohort study*—Give the eligibility criteria, and the sources and methods of selection of participants. Describe methods of follow-up<br>*Case-control study*—Give the eligibility criteria, and the sources and methods of case ascertainment and control selection. Give the rationale for the choice of cases and controls<br>*Cross-sectional study*—Give the eligibility criteria, and the sources and methods of selection of participants	19
		(*b*) *Cohort study*—For matched studies, give matching criteria and number of exposed and unexposed<br>*Case-control study*—For matched studies, give matching criteria and the number of controls per case	
Variables	7	Clearly define all outcomes, exposures, predictors, potential confounders, and effect modifiers. Give diagnostic criteria, if applicable	20
Data sources/ measurement	8*	For each variable of interest, give sources of data and details of methods of assessment (measurement). Describe comparability of assessment methods if there is more than one group	*20, Suppl. Data 1*
Bias	9	Describe any efforts to address potential sources of bias	
Study size	10	Explain how the study size was arrived at	
Quantitative variables	11	Explain how quantitative variables were handled in the analyses. If applicable, describe which groupings were chosen and why	21, 22
Statistical methods	12	(*a*) Describe all statistical methods, including those used to control for confounding	22-23
		(*b*) Describe any methods used to examine subgroups and interactions	23
		(*c*) Explain how missing data were addressed	23
		(*d*) *Cohort study*—If applicable, explain how loss to follow-up was addressed<br>*Case-control study*—If applicable, explain how matching of cases and controls was addressed<br>*Cross-sectional study*—If applicable, describe analytical methods taking account of sampling strategy	
		(*e*) Describe any sensitivity analyses	
**Results**			

Participants	13*	(a) Report numbers of individuals at each stage of study—eg numbers potentially eligible, examined for eligibility, confirmed eligible, included in the study, completing follow-up, and analysed	8
		(b) Give reasons for non-participation at each stage	8, 9
		(c) Consider use of a flow diagram	Figure 1
Descriptive data	14*	(a) Give characteristics of study participants (eg demographic, clinical, social) and information on exposures and potential confounders	8
		(b) Indicate number of participants with missing data for each variable of interest	8
		(c) *Cohort study*—Summarise follow-up time (eg, average and total amount)	
Outcome data	15*	*Cohort study*—Report numbers of outcome events or summary measures over time	*8, 9*
		Case-control study—Report numbers in each exposure category, or summary measures of exposure	
		Cross-sectional study—Report numbers of outcome events or summary measures	
Main results	16	(*a*) Give unadjusted estimates and, if applicable, confounder-adjusted estimates and their precision (eg, 95% confidence interval). Make clear which confounders were adjusted for and why they were included	*9-12*
		(*b*) Report category boundaries when continuous variables were categorized	
		(*c*) If relevant, consider translating estimates of relative risk into absolute risk for a meaningful time period	
Other analyses	17	Report other analyses done—eg analyses of subgroups and interactions, and sensitivity analyses	9-12
**Discussion**			
Key results	18	Summarise key results with reference to study objectives	13, 14
Limitations	19	Discuss limitations of the study, taking into account sources of potential bias or imprecision. Discuss both direction and magnitude of any potential bias	15-16
Interpretation	20	Give a cautious overall interpretation of results considering objectives, limitations, multiplicity of analyses, results from similar studies, and other relevant evidence	13-15
Generalisability	21	Discuss the generalisability (external validity) of the study results	16
**Other information**			
Funding	22	Give the source of funding and the role of the funders for the present study and, if applicable, for the original study on which the present article is based	24

Note: An Explanation and Elaboration article discusses each checklist item and gives methodological background and published examples of transparent reporting. The STROBE checklist is best used in conjunction with this article (freely available on the Web sites of PLoS Medicine at http://www.plosmedicine.org/, Annals of Internal Medicine at http://www.annals.org/, and Epidemiology at http://www.epidem.com/). Information on the STROBE Initiative is available at www.strobe-statement.org.

5 R package environment

For computational reproducibility, the R package versions recorded for the analysis environment are provided in Supplementary Table S1. Packages are listed alphabetically by package name. The source record contained package names and versions only; the R version, operating system, and analysis date should be reported separately if available.

Package	Version	Package	Version	Package	Version
abind	1.4-5	gridtext	0.1.5	purrr	1.0.1
alphavantager	0.1.3	gtable	0.3.3	quadprog	1.5-8
anytime	0.3.9	gtools	3.9.4	Quandl	2.11.0
arrangements	1.1.9	hardhat	1.3.0	quantmod	0.4.22
askpass	1.1	haven	2.5.2	quantreg	5.95
assertthat	0.2.1	HDInterval	0.2.4	quantregForest	1.3-7
backports	1.4.1	highr	0.10	quickmatch	0.2.1
base64enc	0.1-3	Hmisc	5.0-1	R6	2.5.1
beeswarm	0.4.0	hms	1.1.3	RadialMR	1.0
BH	1.81.0-1	htmlTable	2.4.1	ragg	1.2.5
bit	4.0.5	htmltools	0.5.5	randomForest	4.7-1.1
bit64	4.0.5	htmlwidgets	1.6.2	randtoolbox	2.0.4
bitops	1.0-7	httpuv	1.6.9	ranger	0.15.1
blob	1.2.4	httr	1.4.6	rappdirs	0.3.3
bmp	0.3	httr2	0.2.2	rbibutils	2.2.13
brew	1.0-8	ids	1.0.1	rcmdcheck	1.4.0
brio	1.1.3	ieugwasr	0.1.5	RColorBrewer	1.1-3
broom	1.0.4	igraph	1.4.2	Rcpp	1.0.10
bslib	0.4.2	imager	0.42.19	RcppArmadillo	0.12.2.0.0
cachem	1.0.8	infer	1.0.4	RcppEigen	0.3.3.9.3
callr	3.7.3	ini	0.3.1	RcppProgress	0.4.2
car	3.1-2	insight	0.19.1	RcppRoll	0.3.0
carData	3.0-5	interp	1.1-4	Rdpack	2.4
caret	6.0-94	inum	1.0-5	readbitmap	0.1.5
caTools	1.18.2	ipred	0.9-14	readr	2.1.4
CBPS	0.23	isoband	0.2.7	readxl	1.4.2
cellranger	1.1.0	iterators	1.0.14	recipes	1.0.6
checkmate	2.2.0	iterpc	0.4.2	rematch	1.0.1
chk	0.8.1	jpeg	0.1-10	rematch2	2.1.2
CircStats	0.2-6	jquerylib	0.1.4	remotes	2.4.2
cli	3.6.0	jsonlite	1.8.4	reprex	2.0.2
clipr	0.8.0	jtools	2.2.1	reshape	0.8.9
clock	0.6.1	kernlab	0.9-32	reshape2	1.4.4
cobalt	4.5.1	km.ci	0.5-6	rgenoud	5.9-0.3
coin	1.4-2	KMsurv	0.1-5	Rglpk	0.6-5
colorspace	2.1-0	knitr	1.42	riingo	0.3.1
combinat	0.0-8	labeling	0.4.2	rJava	1.0-6
common	1.0.5	laeken	0.5.2	rjson	0.2.21
commonmark	1.9.0	lars	1.3	rlang	1.1.1
CompQuadForm	1.4.3	later	1.3.1	rlemon	0.2.1

Package	Version	Package	Version	Package	Version
conflicted	1.2.0	latticeExtra	0.6-30	rmarkdown	2.21
corrplot	0.92	lava	1.7.2.1	rngWELL	0.10-9
cowplot	1.1.1	lavaan	0.6-15	robustbase	0.95-1
cpp11	0.4.3	lazyeval	0.2.2	ROCR	1.0-11
crayon	1.5.2	lcmm	2.1.0	roxygen2	7.2.3
credentials	1.3.2	leaps	3.1	rprojroot	2.0.3
crosstalk	1.2.0	lhs	1.1.6	rsample	1.1.1
curl	5.0.0	libcoin	1.0-9	rsq	2.5
cvAUC	1.1.4	LiblineaR	2.10-22	rstatix	0.7.2
data.table	1.14.8	lifecycle	1.0.3	rstudioapi	0.14
DBI	1.1.3	listenv	0.9.0	Rtsne	0.16
dbplyr	2.3.2	lme4	1.1-33	rversions	2.1.2
deldir	1.0-6	lmtest	0.9-40	rvest	1.0.3
dendextend	1.17.1	lubridate	1.9.2	sandwich	3.0-2
DEoptimR	1.0-13	magrittr	2.0.3	sass	0.4.6
Deriv	4.1.3	maps	3.4.1	scales	1.2.1
desc	1.4.2	marginaleffects	0.11.1	scatterplot3d	0.3-44
devtools	2.4.5	markdown	1.6	scclust	0.2.3
dfidx	0.0-5	marqLevAlg	2.0.8	selectr	0.4-2
diagram	1.6.5	Matching	4.10-8	sessioninfo	1.2.2
dials	1.2.0	MatchIt	4.5.3	sets	1.0-24
DiceDesign	1.9	mathjaxr	1.6-0	shape	1.4.6
diffobj	0.3.5	MatrixModels	0.5-1	shiny	1.7.4
digest	0.6.31	matrixStats	0.63.0	slam	0.1-50
distances	0.1.9	maxstat	0.7-25	slider	0.3.0
distributional	0.3.2	mboost	2.9-7	smooth	3.2.1
doParallel	1.0.17	memoise	2.0.1	sourcetools	0.1.7-1
dotCall64	1.0-2	MendelianRandomization	0.7.0	sp	1.6-0
downlit	0.4.2	meta	6.2-1	spam	2.9-1
downloader	0.4	metadat	1.2-0	SparseM	1.81
dplyr	1.1.2	metafor	4.2-0	SQUAREM	2021.1
DT	0.27	mime	0.12	stabs	0.6-4
dtplyr	1.3.1	miniUI	0.1.1.1	statmod	1.5.0
dtw	1.23-1	minqa	1.2.5	stepPlr	0.93
e1071	1.7-13	mitools	2.4	stringi	1.7.12
egg	0.4.5	mlbench	2.1-3.1	stringr	1.5.0
elasticnet	1.3	MLeval	0.3	strucchange	1.5-3
ellipse	0.4.5	mlogit	1.1-1	SuperLearner	2.0-28
ellipsis	0.3.2	mnormt	2.1.1	survey	4.2-1
emmeans	1.8.5	modeldata	1.1.0	survminer	0.4.9
estimability	1.4.1	modelenv	0.1.1	survMisc	0.5.6
evaluate	0.21	ModelMetrics	1.2.2.2	sys	3.4.1
exactRankTests	0.8-35	modelr	0.1.11	systemfonts	1.0.4
factoextra	1.0.7	modeltools	0.2-23	testthat	3.1.8
FactoMineR	2.8	mr.raps	0.2	texreg	1.38.6

Package	Version	Package	Version	Package	Version
fansi	1.0.4	MRInstruments	0.3.2	textshaping	0.3.6
farver	2.1.1	MRMix	0.1.0	TH.data	1.1-2
fastmap	1.1.1	MRPRESSO	1.0	this.path	1.4.0
fields	14.1	multcomp	1.4-23	tibble	3.2.1
flashClust	1.01-2	multcompView	0.1-9	tidymodels	1.1.0
flsa	1.5.2	munsell	0.5.0	tidyquant	1.0.7
fmsb	0.7.5	mvtnorm	1.1-3	tidyr	1.3.0
fmtr	1.5.9	nloptr	2.0.3	tidyselect	1.2.0
fontawesome	0.5.1	nnls	1.4	tidyverse	2.0.0
forcats	1.0.0	nortest	1.0-4	tiff	0.1-11
foreach	1.5.2	numDeriv	2016.8-1.1	timechange	0.2.0
forecast	8.21	OddsPlotty	1.0.2	timeDate	4022.108
Formula	1.2-5	openssl	2.0.6	timetk	2.8.3
fracdiff	1.5-2	optmatch	0.10.6	tinytex	0.45
fs	1.6.2	optweight	0.2.5	translations	4.2.2
furrr	0.3.1	ordinalForest	2.4-3	tseries	0.10-54
future	1.32.0	osqp	0.6.0.8	tsfeatures	1.1
future.apply	1.10.0	padr	0.6.2	TTR	0.24.3
gam	1.22-2	pander	0.6.5	tune	1.1.1
gargle	1.4.0	parallelly	1.35.0	TwoSampleMR	0.5.6
gbm	2.1.8.1	parsnip	1.1.0	tzdb	0.3.0
gdata	2.19.0	party	1.3-13	urca	1.3-3
generics	0.1.3	partykit	1.2-20	urlchecker	1.0.1
gert	1.9.2	patchwork	1.1.2	usethis	2.1.6
GGally	2.1.2	pbapply	1.7-0	utf8	1.2.3
ggbeeswarm	0.7.2	pbivnorm	0.6.0	uuid	1.1-0
ggdist	3.2.1	pbkrtest	0.5.2	vcd	1.4-11
gghalves	0.1.4	penalizedLDA	1.1	vctrs	0.6.2
ggnewscale	0.4.8	PerformanceAnalytics	2.0.4	verification	1.42
ggplot2	3.4.2	pillar	1.9.0	VIM	6.2.2
ggpubr	0.6.0	pkgbuild	1.4.0	vipor	0.4.5
ggradar	0.2	pkgconfig	2.0.3	viridis	0.6.3
ggrepel	0.9.3	pkgdown	2.0.7	viridisLite	0.4.2
ggsci	3.0.0	pkgload	1.3.2	vroom	1.6.3
ggsignif	0.6.4	plotly	4.10.1	waldo	0.5.0
ggtext	0.1.2	plotROC	2.3.0	warp	0.2.0
ggthemes	4.2.4	plyr	1.8.8	WeightIt	0.14.0
gh	1.4.0	png	0.1-8	whisker	0.4.1
gitcreds	0.1.2	polynom	1.4-1	withr	2.5.0
glmnet	4.1-7	pracma	2.4.2	workflows	1.1.3
globals	0.16.2	praise	1.0.0	workflowsets	1.0.1
glue	1.6.2	prettyunits	1.1.1	xfun	0.39
gmodels	2.18.1.1	pROC	1.18.0	xlsx	0.6.5
gmp	0.7-1	processx	3.8.1	xlsxjars	0.6.1
googleAuthR	2.0.1	prodlim	2023.03.31	XML	3.99-0.14
googledrive	2.1.0	profvis	0.3.8	xml2	1.3.4

Package	Version	Package	Version	Package	Version
googlesheets4	1.1.0	progress	1.2.2	xopen	1.0.0
gower	1.0.1	progressr	0.13.0	xtable	1.8-4
GPfit	1.0-8	promises	1.2.0.1	xts	0.13.1
gplots	3.1.3	proxy	0.4-27	yaml	2.3.7
greybox	1.0.8	ps	1.7.5	yardstick	1.2.0
gridExtra	2.3	pscl	1.5.5.1	zip	2.3.0
gridSVG	1.7-5	psych	2.3.3	zoo	1.8-12
