# Supplementary material for: Asymmetric plantar temperature downshifts are associated with atrial fibrillation and thromboembolic events: an observational post hoc analysis of the SmartPreventDiabeticFeet study
Source: Commun Med (Lond). 2026 Jul 31;6:424. doi: 10.1038/s43856-026-01811-3 (PMC13427832; doi:10.1038/s43856-026-01811-3)
Supplement: Supplementary file 2 — Description of Additional Supplementary Files [file 43856_2026_1811_MOESM2_ESM.docx]

Description of Additional Supplementary Files

**File name:** Supplementary Data 1

**Description:** Source data underlying Figures 1–4. This Excel workbook contains the numerical source data underlying the graphs, charts and plotted data shown in Figures 1–4. Data are organized by figure and, where applicable, by figure panel. The workbook includes source data for the study timeline and cohort flow, post-hoc plantar temperature downshift classification, plantar sensor metadata, representative longitudinal temperature recordings, temperature-variability clustering, silhouette-based cluster validation, Kaplan–Meier curve data, patient-level event timeline plotting data, PIRI symmetry/asymmetry group summaries, composite AF/thromboembolic outcome counts and statistical test summaries. Patient identifiers are study-specific pseudonymous identifiers and do not contain direct personal identifiers.
